# CWL-Airflow: a lightweight pipeline manager supporting Common Workflow Language

**DOI:** 10.1093/gigascience/giz084

**Published:** 2019-07-18

**Authors:** Michael Kotliar, Andrey V Kartashov, Artem Barski

**Affiliations:** 1 Division of Allergy and Immunology, Cincinnati Children's Hospital Medical Center and Department of Pediatrics, College of Medicine, University of Cincinnati, Cincinnati, OH, USA; 2 Division of Human Genetics, Cincinnati Children's Hospital Medical Center and Department of Pediatrics, College of Medicine, University of Cincinnati, Cincinnati, OH, USA

**Keywords:** Common Workflow Language, workflow manager, pipeline manager, Airflow, reproducible data analysis, workflow portability

## Abstract

**Background:**

Massive growth in the amount of research data and computational analysis has led to increased use of pipeline managers in biomedical computational research. However, each of the >100 such managers uses its own way to describe pipelines, leading to difficulty porting workflows to different environments and therefore poor reproducibility of computational studies. For this reason, the Common Workflow Language (CWL) was recently introduced as a specification for platform-independent workflow description, and work began to transition existing pipelines and workflow managers to CWL.

**Findings:**

Herein, we present CWL-Airflow, a package that adds support for CWL to the Apache Airflow pipeline manager. CWL-Airflow uses CWL version 1.0 specification and can run workflows on stand-alone MacOS/Linux servers, on clusters, or on a variety of cloud platforms. A sample CWL pipeline for processing of chromatin immunoprecipitation sequencing data is provided.

**Conclusions:**

CWL-Airflow will provide users with the features of a fully fledged pipeline manager and the ability to execute CWL workflows anywhere Airflow can run—from a laptop to a cluster or cloud environment. CWL-Airflow is available under Apache License, version 2.0 (Apache-2.0), and can be downloaded from https://barski-lab.github.io/cwl-airflow, https://scicrunch.org/resolver/RRID:SCR_017196.

## Background

Modern biomedical research has seen a remarkable increase in the production and computational analysis of large datasets, leading to an urgent need to share standardized analytical techniques. However, of the >100 computational workflow systems used in biomedical research, most define their own specifications for computational pipelines [1, 2]. Furthermore, the evolving complexity of computational tools and pipelines makes it nearly impossible to reproduce computationally heavy studies or to repurpose published analytical workflows. Even when the tools are published, the lack of a precise description of the operating system environment and component software versions can lead to inaccurate reproduction of the analyses—or analyses failing altogether when executed in a different environment. To ameliorate this situation, a team of researchers and software developers formed the Common Workflow Language (CWL) working group [3] with the intent of establishing a specification for describing analysis workflows and tools in a way that makes them portable and scalable across a variety of software and hardware environments. The CWL specification provides a set of formalized rules that can be used to describe each command line tool and its parameters, and optionally a container (e.g., a Docker [4] or Singularity [5] image) with the tool already installed. CWL workflows are composed of ≥1 such command line tools. Thus, CWL provides a description of the working environment and version of each tool, how the tools are “connected” together, and what parameters were used in the pipeline. Researchers using CWL are then able to deposit descriptions of their tools and workflows into a repository (e.g., dockstore.org) upon publication, thus making their analyses reusable by others.

After version 1.0 of the CWL standard [6] and the reference executor, cwltool, were finalized in 2016, developers began adapting the existing pipeline managers to use CWL. For example, companies such as Seven Bridges Genomics and Curoverse are developing the commercial platforms Rabix [7] and Arvados [8], whereas academic developers (e.g., Galaxy [9], Toil [10], and others) are adding CWL support to their pipeline managers (see Discussion).

Airflow [11] is a lightweight workflow manager initially developed by AirBnB, which has now graduated from Apache Incubator, and is available under a permissive Apache license. Airflow executes each workflow as a directed acyclic graph (DAG) of tasks. The tasks are usually atomic and are not supposed to share any resources with each other; therefore, they can be run independently. The DAG describes relationships between the tasks and defines their order of execution. The DAG objects are initiated from Python scripts placed in a designated folder. Airflow has a modular architecture and can distribute tasks to an arbitrary number of workers and across multiple servers while adhering to the task sequence and dependencies specified in the DAG. Unlike many of the more complicated platforms, Airflow imposes little overhead, is easy to install, and can be used to run task-based workflows in various environments ranging from stand-alone desktops and servers to Amazon or Google cloud platforms. It also scales horizontally on clusters managed by Apache Mesos [12] and may be configured to send tasks to the Celery [13] task queue. Herein, we present an extension of Airflow, allowing it to run CWL-based pipelines. Altogether, this gives us a lightweight workflow management system with full support for CWL, the most promising scientific workflow description language.

## Methods

**Figure 1:**
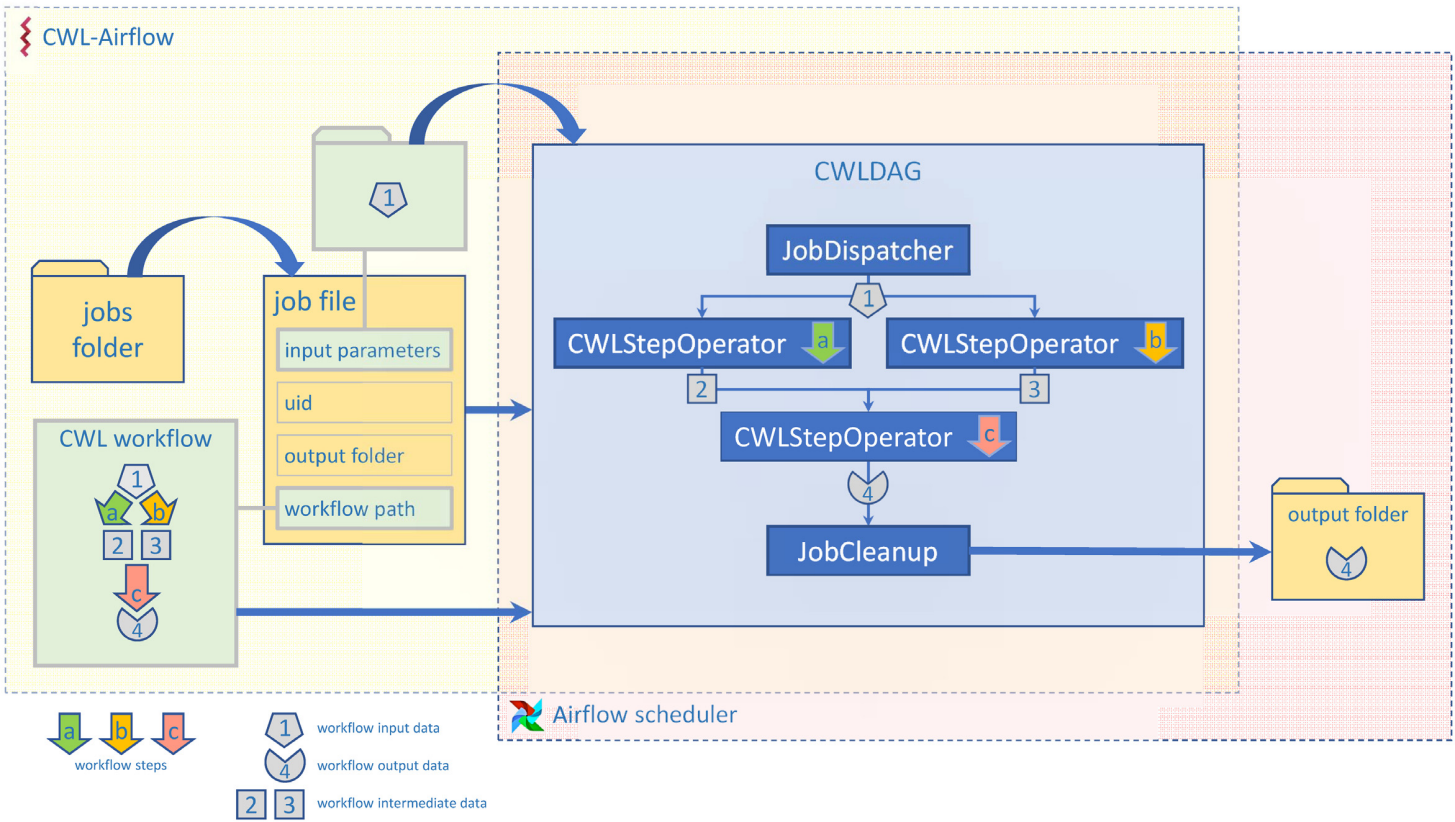
CWL-Airflow diagram. The job file contains information about the CWL workflow and inputs. CWL-Airflow creates a CWLDAG-class instance on the basis of the workflow structure and executes it in Airflow. The results are saved to the output folder.

The CWL-Airflow package extends Airflow's functionality with the ability to parse and execute workflows written with the CWL version 1.0 (v1.0) specification [6]. CWL-Airflow can be easily integrated into the Airflow scheduler logic as shown in the structure diagram in Fig. 1. The Apache Airflow code is extended with a Python package that defines 4 basic classes—JobDispatcher, CWLStepOperator, JobCleanup, and CWLDAG. Additionally, the automatically generated cwl_dag.py script is placed in the DAGs folder. While periodically loading DAGs from the DAGs folder, the Airflow scheduler runs the cwl_dag.py script and creates DAGs on the basis of the available jobs and corresponding CWL workflow descriptor files.

In order to run a CWL workflow in Airflow, a file describing the job should be placed in the jobs folder (Fig. 1). The jobs are described by a file in JSON or YAML format that includes workflow-specific input parameters (e.g., input file locations) and 3 mandatory fields: workflow (absolute path to the CWL descriptor file to be run with this job), output_folder (absolute path to the folder where all the output files should be moved after successful pipeline execution), and uid (unique identifier for the run). CWL-Airflow parses every job file from the jobs folder, loads the corresponding CWL workflow descriptor file, and creates a CWLDAG-class instance on the basis of the workflow structure and input parameters provided in the job file. The uid field from the job file is used to identify the newly created CWLDAG-class instance.

CWLDAG is a class for combining the tasks into a DAG that reflects the CWL workflow structure. Every CWLStepOperator task corresponds to a workflow step and depends on others on the basis of the workflow step inputs and outputs. This implements dataflow principles and architecture that are missing in Airflow. Additionally, the JobDispatcher and JobCleanup tasks are added to the DAG. JobDisptacher is used to serialize the input parameters from the job file and provide the pipeline with the input data; JobCleanup returns the calculated results to the output folder. When the Airflow scheduler executes the pipeline from the CWLDAG, it runs the workflow with the structure identical to the CWL descriptor file used to create this graph.

**Figure 2:**
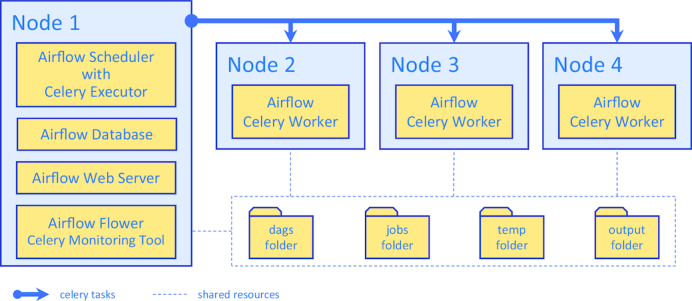
Structure diagram for scaling out CWL-Airflow with a Celery cluster of 4 nodes. Node 1 runs the Airflow database to save task metadata and the Airflow scheduler with the Celery executor to submit tasks for processing to the Airflow celery workers on Nodes 2, 3, and 4. The Airflow and Flower (Celery) web servers allow for monitoring and controlling of the task execution process. All nodes have shared access to the dags, jobs, temp, and output folders.

Although running CWL-Airflow on a single node may be sufficient in most cases, it is worth switching to the multi-node configuration (Fig. 2) for computationally intensive pipelines. Airflow uses the Celery task queue to distribute processing over multiple nodes. Celery provides the mechanisms for queueing and assigning tasks to multiple workers, whereas the Airflow scheduler uses Celery executor to submit tasks to the queue. The Celery system helps not only to balance the load over the different machines but also to define task priorities by assigning them to the separate queues.

**Figure 3:**
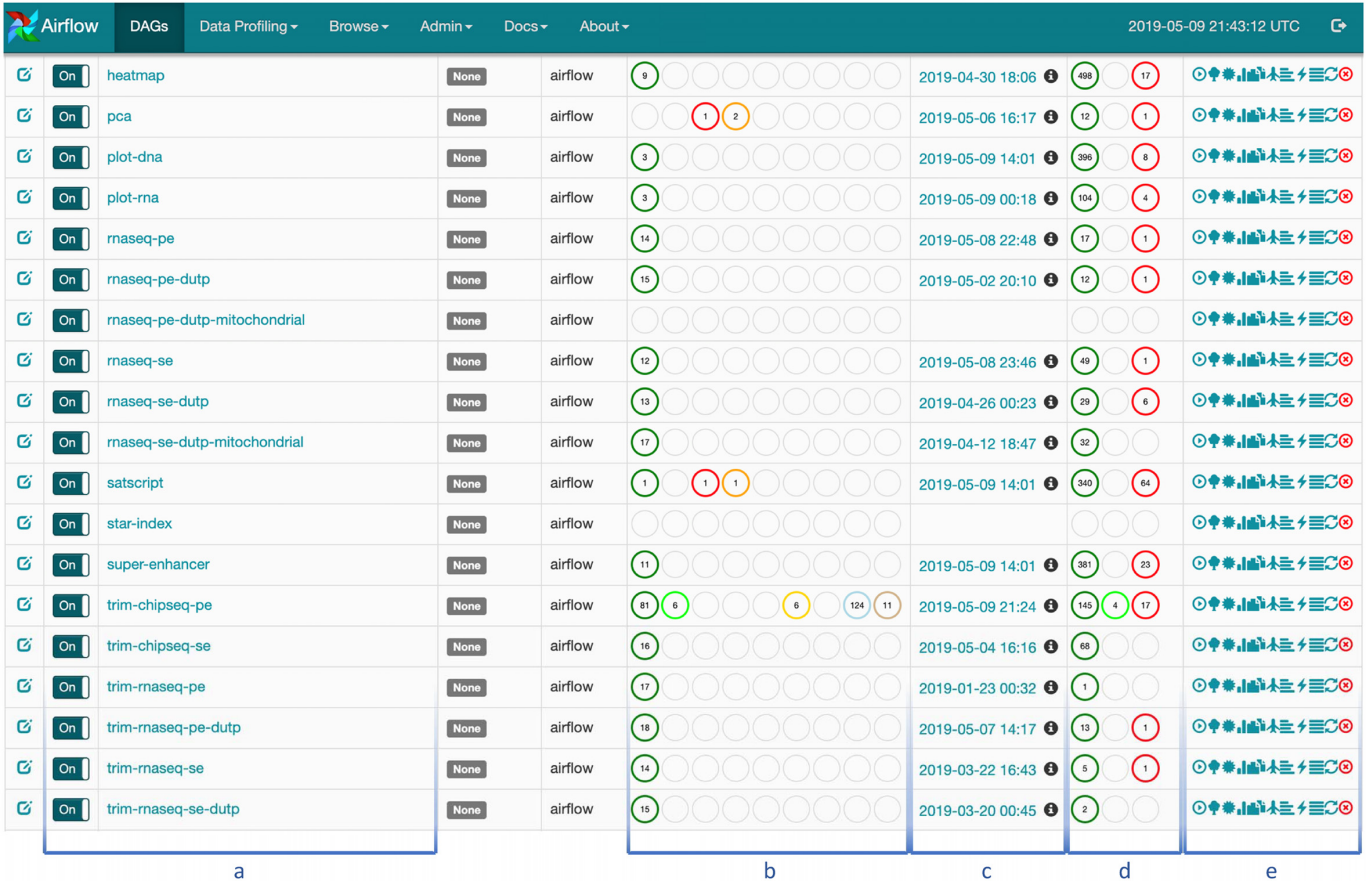
Airflow web interface. The DAGs tab shows the list of the available pipelines (a) and their latest execution dates (c) and number of active, succeeded, and failed runs (d) and workflow step statuses (b). The buttons on the right (e) allow a user to control pipeline execution and obtain additional information on the current workflow and its steps.

**Figure 4:**
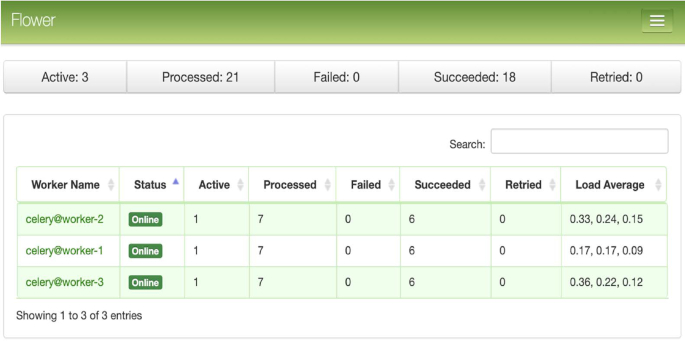
Dashboard of the Celery monitoring tool Flower. Shown are the 3 Celery workers, their current status, and load information.

An example of a CWL-Airflow Celery cluster of 4 nodes is shown in Fig. 2. The tasks are submitted to the queue by Node 1 and executed by any of the 3 workers (Nodes 2, 3, and 4). Node 1 runs 2 mandatory components—the Airflow database and scheduler. The latter schedules the task execution by adding tasks to the queue. All Celery workers are subscribed to the same task queue. Whenever an arbitrary worker pulls a new task from the queue, it runs the task and returns the execution results. For the sequential steps, the Airflow scheduler submits the next tasks to the queue. During the task execution, intermediate data are kept in the temp folder. Upon successful pipeline completion, all output files are moved to the output folder. Both the temp and output folders, as well as the dags and jobs folders, are shared among all the nodes of the cluster. Optionally, Node 1 can also run the Airflow web server (Fig. 3) and the Celery monitoring tool Flower (Fig. 4) to provide users with the pipeline execution details.

## Results

### ChIP-Seq analysis with CWL-Airflow

**Figure 5:**
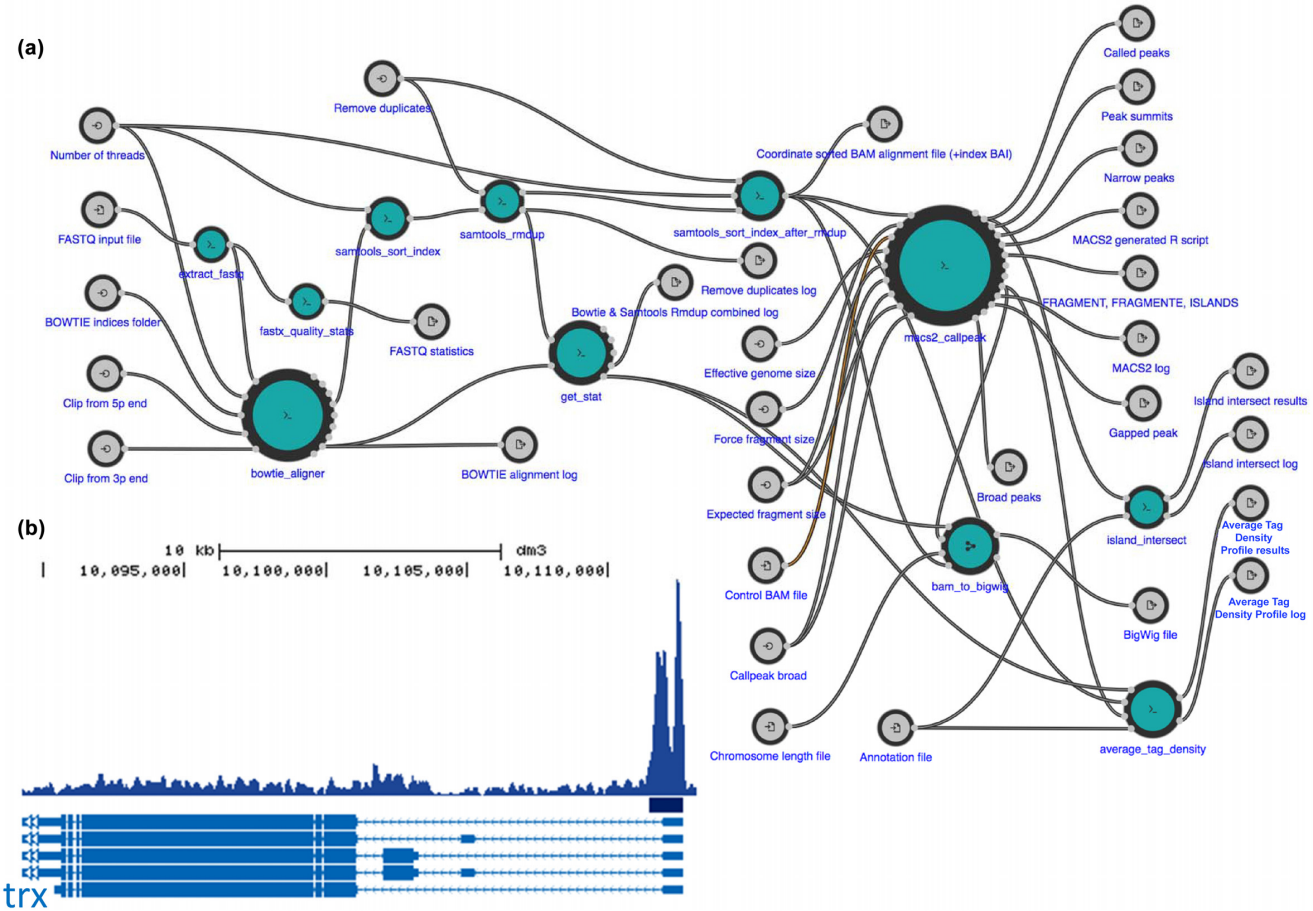
Using CWL-Airflow for analysis of ChIP-Seq data. (a) ChIP-Seq data analysis pipeline visualized by Rabix Composer. (b) *Drosophila melanogaster*embryo histone 3, lysine 4 trimethylation (H3K4me3) ChIP-Seq data (SRR1198790) were processed by our pipeline and CWL-Airflow. University of California Santa Criz genome browser view of tag density and peaks at the *trx* gene is shown. View via the Common Workflow Language Viewer permalink here: https://w3id.org/cwl/view/git/f28d47bd0911e5e7210c4dc83f75653a1e0297c9/biowardrobe_chipseq_se.cwl. ATDP: Average Tag Density Profile.

As an example, we used a workflow for basic analysis of chromatin immunoprecipitation sequencing (ChIP-Seq) data (Fig.   5, Research object: Additional file 1). This workflow is a CWL version of a Python pipeline from BioWardrobe [14, 15]. It starts by using BowTie [16] to perform alignment to a reference genome, resulting in an unsorted SAM file. The SAM file is then sorted and indexed with SAMtools [17] to obtain a BAM file and a BAI index. Next MACS2 [18] is used to call peaks and to estimate fragment size. In the last few steps, the coverage by estimated fragments is calculated from the BAM file and is reported in bigWig format (Fig. 5). The pipeline also reports statistics, such as read quality, peak number, and base frequency, and other troubleshooting information using tools such as FASTX-Toolkit [19] and BamTools [20]. The directions for how to run a sample pipeline can be found on the CWL-Airflow web page [21]. Execution time in CWL-Airflow was similar to that of reference implementation (Table 1).

**Table 1:** CWL-Airflow and cwltool mean execution time

Pipeline	Mean ± SEM (seconds), n = 3		
	CWL-Airflow		Cwltool
	1 Node, 1 workflow at a time	3 Nodes, 3 workflows at a time	1 Node, 1 workflow at a time
BioWardrobe ChIP-Seq Workflow	1,141 ± 18	1,231 ± 3	955 ± 1
ENCODE ChIP-Seq Mapping Workflow	3,784 ± 10	3,824 ± 28	3,245 ± 7

ChIP-Seq, chromatin immunoprecipitation sequencing; CWL, common workflow language; SEM, standard error of the mean.

The CWL-Airflow package includes 2 additional demonstration workflows: (i) an identification of super-enhancers [22] and (ii) a simplified version of the Xenbase [23] RNA sequencing (RNA-Seq) pipeline. More pipelines can be found elsewhere. In particular, BioWardrobe's [14] pipelines for analysis of single-read and paired-end ChIP-Seq and of stranded and un-stranded, single and paired RNA-Seq are available on GitHub [24]. Additional collections of tools are available in Rabix Composer [7], a graphical CWL editor from Seven Bridges and at the Dockstore [25].

### Portability of CWL analyses

The key promise of CWL is the portability of analyses. Portability refers to the ability to seamlessly run a containerized CWL pipeline developed for one CWL platform on another CWL platform, allowing users to easily share computational workflows. To check whether CWL-Airflow can use pipelines developed by others, we downloaded an alternative workflow for the analysis of ChIP-Seq data developed by the Encyclopedia of DNA Elements (ENCODE) Data Coordination Center [26, 27] using a test dataset (CCAAT/enhancer-binding protein β [CEBPB] ChIP-Seq in A549 cells, ENCODE accession: ENCSR000DYI). CWL-Airflow was able to run the pipeline and produced results identical to those obtained with the reference cwltool. The execution time is shown in Table 1. Notably, running the tested pipelines on the single-node CWL-Airflow system increased execution time by 18%, whereas running them on the 3-node CWL-Airflow cluster reduced execution time by 41% per workflow compared to the reference cwltool. These results confirm that CWL-Airflow complies with the CWL specification, supports portability, and performs analysis in a reproducible manner. Additional testing of pipeline portability is currently being conducted as a part of the Global Alliance for Genomics and Health (GA4GH) workflow portability challenge [28].

### CWL-Airflow in multi-node configuration with Celery executor

To demonstrate the use of CWL-Airflow in a multi-node configuration, we set up a Celery cluster of 3 nodes with 4 CPUs and 94 GB of RAM each, with each node running an instance of the Airflow Celery worker. Tasks were queued for execution by the Airflow scheduler that was launched on the first node. Communication between the Celery workers was managed by the message queueing service RabbitMQ. RabbitMQ, as well as the Airflow database and web server, were run on the first node. Executing the 2 tested pipelines on the Airflow Celery cluster demonstrated only a slight slowdown on a per-run basis (Table 1).

## Discussion

CWL-Airflow is one of the first pipeline managers supporting version 1.0 of the CWL standard and provides a robust and user-friendly interface for executing CWL pipelines. Unlike more complicated pipeline managers, the installation of Airflow and the CWL-Airflow extension can be performed with a single pip install command. Compared to the competing pipeline managers, Airflow has multiple advantages (Table 2). Specifically, Airflow provides a wide range of tools for managing the workflow execution process, such as pausing and resuming the workflow execution, stopping and restarting the individual workflow steps, restarting the workflow from a certain step, and skipping part of the workflow by updating the states of the specific steps from a web-based GUI. Similar to other workflow management systems, Airflow can run on clusters and the major cloud services. Unlike some of the workflow executors, Airflow supports both Docker and Singularity containerization technologies. The latter is particularly important because many clusters do not allow the use of Docker for security reasons.

**Table 2:** Comparison of the open source workflow managers and engines with existing or planned support for CWL

Feature	Airflow and CWL-Airflow			Rabix			Toil			Cromwell			REANA			Galaxy			Arvados			CWLEXEC		
Software installation complexity	Single Python package			JAR			Single Python package			JAR			Group of Python packages			Group of Python packages			Multiple components for minimum 7 nodes system			JAR		
				Electron application												node.js application								
License type	Apache License v2.0			Apache License v2.0			Apache License v2.0			BSD-3-Clause			MIT License			Academic Free License v3.0			Apache License v2.0, AGPL v3.0, CC-BY-SA v3.0			Apache License v2.0		
Workflow description language	CWL v1.0			CWL v1.0			CWL v1.0			CWL v1.0			CWL v1.0			XML tool file			CWL v1.0			CWL v1.0		
	Python code						WDL v1.0			WDL v1.0			Serial			JSON workflow file								
							Python code						Yadage											
Docker containerization	**+**			**+**			**+**			**+**			**+**			**+**			**+**			**+**		
Singularity containerization	**+**			**−**			**+**			**+**			**−**			**+**			**−**			**−**		
Cloud/cluster processing	**+**			**−**			**+**			**+**			**+**			**+**			**+**			**+**		
Workflow execution load balancing^1^	**+**			**−**			**+**			**+**			**+**			**+**			**+**			**+**		
Parallel workflow step execution	**+**			**+**			**+**			**+**			**+**			**+**			**+**			**+**		
	GUI	REST API	CLI	GUI	REST API	CLI	GUI	REST API	CLI	GUI	REST API	CLI	GUI	REST API	CLI	GUI	REST API	CLI	GUI	REST API	CLI	GUI	REST API	CLI
Add new workflow^2^	**−**	**−**	**+**	**+**	∅	**+**	∅	∅	**+**	∅	**+**	**+**	∅	**+**	**+**	**+**	**+**	∅	**+**	**+**	**+**	∅	∅	**+**
Set workflow inputs^3^	**−**	**+**	**+**	**+**	∅	**+**	∅	∅	**+**	∅	**+**	**+**	∅	**+**	**+**	**+**	**+**	∅	**+**	**+**	**+**	∅	∅	**+**
Start/stop workflow execution	**+**	**+**	**+**	**+**	∅	**+**	∅	∅	**+**	∅	**+**	**+**	∅	**+**	**+**	**+**	**+**	∅	**+**	**+**	**+**	∅	∅	**+**
Manage workflow execution process^4^	**+**	**+**	**+**	**−**	∅	**−**	∅	∅	**+**	∅	**−**	**−**	∅	**+**	**+**	**+**	**+**	∅	**−**	**+**	**+**	∅	∅	**+**
Get execution results of the specific workflow^5^	**+**	**−**	**−**	**+**	∅	**−**	∅	∅	**+**	∅	**+**	**−**	∅	**+**	**+**	**+**	**+**	∅	**+**	**+**	**+**	∅	∅	**−**
View workflow execution logs	**+**	**−**	**+**	**+**	∅	**+**	∅	∅	**+**	∅	**+**	**+**	∅	**+**	**+**	**+**	**+**	∅	**+**	**+**	**+**	∅	∅	**+**
View workflow execution history and statistics	**+**	**+**	**+**	**−**	∅	**−**	∅	∅	**+**	∅	**+**	**−**	∅	**+**	**+**	**+**	**+**	∅	**+**	**+**	**+**	∅	∅	**+**

+, Present; –, absent; ∅, not applicable; AGPL: Affero General Public License; BSD: Berkely Source Distribution; CC-BY-SA: Creative Commons Attribution-Share-Alike; CLI, command line interface; GUI, graphical user interface; MIT: Massachusetts Institute of Technology; REST API, representational state transfer application program interface; WDL, workflow description language.

^1^Assign workflow steps to the different pools and queues; use other resource utilization algorithms provided by the computing environment.

^2^Load the workflow from the file; create the workflow by combining the steps in GUI.

^3^Set the path to the job file; set input values through the GUI or CLI.

^4^Pause/resume workflow execution process; manually restart workflow steps.

^5^Get output file locations by the workflow ID, step ID, execution date, or other identifiers.

Unlike most of the other workflow managers, Airflow provides a convenient, web-based GUI that allows a user to monitor and control the pipeline execution. Within this web interface, a user can easily track the workflow execution history and collect and visualize statistics from multiple workflow runs. Similar to some of the other pipeline managers, Airflow provides a REST API that allows a user to access its functionality through the dedicated end points. The API can be used by other software to communicate with the Airflow system.

Airflow supports parallel workflow step execution. Step parallelization can be convenient when the workflow complexity is not high and the computational resources are not limited. However, when running multiple workflows, especially on a multi-node system, it becomes reasonable to limit parallelism and balance load over the available computing resources. Besides the standard load-balancing algorithms provided by the computing environment, Airflow supports pools and queues that allow for even distribution of tasks among multiple nodes.

Addition of the CWL capability to Airflow has made it more convenient for scientific computing, in which the users are more interested in the flow of data than the tasks being executed. Although Airflow itself (and most of the pipeline managers [28]) only define workflows as sequences of steps to be executed (e.g., DAGs), the CWL description of inputs and outputs leads to better representation of data flow, which allows for a better understanding of data dependencies and produces more readable workflows.

Furthermore, as one of the most lightweight pipeline managers, Airflow contributes only a small amount of overhead to the overall execution of a computational pipeline (Table 1). We believe that this overhead is an advantageous exchange for (i) Airflow's ability to monitor and control workflow execution and (ii) CWL's enabling of better reproducibility and portability of biomedical analyses. In summary, CWL-Airflow will provide users with the ability to execute CWL workflows anywhere Airflow can run—from a laptop to a cluster or cloud environment.

## Abbreviations

AGPL: Affero General Public License; ATDP: Average Tag Density Profile; BSD: Berkely Source Distribution; CC-BY-SA: Creative Commons Attribution-Share-Alike; CEBPB: CCAAT/enhancer-binding protein β; ChIP-Seq: chromatin immunoprecipitation sequencing; CLI: command line interface; CPU: central processing unit; CWL: Common Workflow Language; DAG: directed acyclic graph; ENCODE: Encyclopedia of DNA Elements; GUI: graphical user interface; JSON: JavaScript Object Notation; MACS: Model-based Analysis of ChIP-Seq; MIT: Massachusetts Institute of Technology; RAM: random access memory; REST API: representational state transfer application program interface; RNA-Seq: RNA sequencing.

## Availability of supporting data and materials

No new datasets or materials were generated. The source code is available under Apache license v2.0 (Apache-2.0) and can be downloaded from https://barski-lab.github.io/cwl-airflow, http://doi.org/10.5281/zenodo.2852870, and RRID: SCR_01 7196. Snapshots and Research Object bundles from the example workflow are also available in the *GigaScience* GigaDB repository [29]. Project name: CWL-Airflow Project home page: https://barski-lab.github.io/cwl-airflow/ Operating system: macOS/Linux Programming language: Python Other requirements: Docker License: Apache license v2.0 (Apache-2.0) RRID: SCR_017196, http://doi.org/10.5281/zenodo.2852870.

## Competing interests

A.V.K. and A.B. are co-founders of Datirium, LLC. Datirium, LLC, provides bioinformatics software support services.

## Funding

The project was supported in part by the Center for Clinical & Translational Research and Training (National Institutes of Health CTSA grant UL1TR001425) and by the NIH NIGMS New Innovator Award to A.B. (DP2GM119134). The funders had no role in study design, data collection and analysis, decision to publish, or preparation of the manuscript.

## Authors' contributions

A.V.K. and A.B. conceived the project; A.V.K. and M.K. wrote the software; and M.K., A.V.K., and A.B. wrote and reviewed the manuscript.

## Supplementary Material

giz084_GIGA-D-19-00044_Original_Submission

giz084_GIGA-D-19-00044_Revision_1

giz084_GIGA-D-19-00044_Revision_2

giz084_Response_to_Reviewer_Comments_Original_Submission

giz084_Response_to_Reviewer_Comments_Revision_1

giz084_Reviewer_1_Report_Original_Submission Niels Drost -- 3/20/2019 Reviewed

giz084_Reviewer_2_Report_Original_Submission Samuel Lampa, PhD -- 4/4/2019 Reviewed

giz084_Reviewer_2_Report_Revision_1 Samuel Lampa, PhD -- 6/10/2019 Reviewed
