# Supplementary material for: CWL-Airflow: a lightweight pipeline manager supporting Common Workflow Language
Source: Gigascience. 2019 Jul 18;8(7):giz084. doi: 10.1093/gigascience/giz084 (PMC6639121; doi:10.1093/gigascience/giz084)
Supplement: giz084_GIGA-D-19-00044_Original_Submission [file giz084_giga-d-19-00044_original_submission.pdf]

GigaScience

CWL-Airflow: a lightweight pipeline manager supporting Common Workflow Language

--Manuscript Draft--

**Manuscript Number:**	GIGA-D-19-00044
**Full Title:**	CWL-Airflow: a lightweight pipeline manager supporting Common Workflow Language
**Article Type:**	Technical Note
**Funding Information:**	National Institute of General Medical Sciences (DP2GM119134) — Dr Artem Barski
**Abstract:**	Background<br><br>Massive growth in the amount of research data and computational analysis has led to increased utilization of pipeline managers in biomedical computational research. However, each of more than 100 such managers uses its own way to describe pipelines, leading to difficulty porting workflows to different environments and therefore poor reproducibility of computational studies. For this reason, the Common Workflow Language (CWL) was recently introduced as a specification for platform-independent workflow description, and work began to transition existing pipelines and workflow managers to CWL.<br><br>Findings<br><br>Here, we present CWL-Airflow, an extension for the Apache Airflow pipeline manager supporting CWL. CWL-Airflow utilizes CWL v1.0 specification and can be used to run workflows on standalone MacOS/Linux servers, on clusters, or on variety cloud platforms. A sample CWL pipeline for processing of ChIP-Seq data is provided.<br><br>Conclusions<br><br>CWL-Airflow will provide users with the features of a fully-fledged pipeline manager and an ability to execute CWL workflows anywhere Airflow can run—from a laptop to cluster or cloud environment.<br><br>Availability<br><br>CWL-Airflow is available under Apache license v.2 and can be downloaded from https://barski-lab.github.io/cwl-airflow.
**Corresponding Author:**	Artem Barski<br>Cincinnati Children's Hospital Medical Center<br>UNITED STATES
**Corresponding Author Secondary Information:**	
**Corresponding Author's Institution:**	Cincinnati Children's Hospital Medical Center
**Corresponding Author's Secondary Institution:**	
**First Author:**	Michael Kotliar, MS
**First Author Secondary Information:**	
**Order of Authors:**	Michael Kotliar, MS<br>Andrey V. Kartashov, MS<br>Artem Barski, PhD
**Order of Authors Secondary Information:**	
**Additional Information:**	

Powered by Editorial Manager® and ProduXion Manager® from Aries Systems Corporation

Question	Response
Are you submitting this manuscript to a special series or article collection?	No
**Experimental design and statistics**<br><br>Full details of the experimental design and statistical methods used should be given in the Methods section, as detailed in our Minimum Standards Reporting Checklist. Information essential to interpreting the data presented should be made available in the figure legends.<br><br>Have you included all the information requested in your manuscript?	Yes
**Resources**<br><br>A description of all resources used, including antibodies, cell lines, animals and software tools, with enough information to allow them to be uniquely identified, should be included in the Methods section. Authors are strongly encouraged to cite Research Resource Identifiers (RRIDs) for antibodies, model organisms and tools, where possible.<br><br>Have you included the information requested as detailed in our Minimum Standards Reporting Checklist?	Yes
**Availability of data and materials**<br><br>All datasets and code on which the conclusions of the paper rely must be either included in your submission or deposited in publicly available repositories (where available and ethically appropriate), referencing such data using a unique identifier in the references and in the "Availability of Data and Materials" section of your manuscript.	Yes

Have you have met the above requirement as detailed in our Minimum Standards Reporting Checklist?	

Powered by Editorial Manager® and ProduXion Manager® from Aries Systems Corporation

Manuscript+figures
Click here to download Manuscript cwl_airflow-Feb13-Giga.docx

CWL-Airflow: a lightweight pipeline manager supporting Common Workflow Language

Michael Kotliar[1,*], Andrey V. Kartashov[1,*], and Artem Barski[1,2,#]

[1]Division of Allergy and Immunology, [2]Division of Human Genetics, Cincinnati Children's Hospital Medical Center and Department of Pediatrics, College of Medicine, University of Cincinnati, Cincinnati, OH

*Joint first author; #To whom correspondence should be addressed: Artem.Barski@cchmc.org.

Emails:

MK: michael.kotliar@cchmc.org

AVK: andrey.kartashov@cchmc.org

AB: Artem.barski@cchmc.org

Short title: CWL-Airflow pipeline manager

Abstract

Background: *Massive growth in the amount of research data and computational analysis has led to increased utilization of pipeline managers in biomedical computational research. However, each of more than 100 such managers uses its own way to describe pipelines, leading to difficulty porting workflows to different environments and therefore poor reproducibility of computational studies. For this reason, the Common Workflow Language (CWL) was recently introduced as a specification for platform-independent workflow description, and work began to transition existing pipelines and workflow managers to CWL.*

Findings: *Here, we present CWL-Airflow, an extension for the Apache Airflow pipeline manager supporting CWL. CWL-Airflow utilizes CWL v1.0 specification and can be used to run workflows on standalone MacOS/Linux servers, on clusters, or on variety cloud platforms. A sample CWL pipeline for processing of ChIP-Seq data is provided.*

Conclusions: *CWL-Airflow will provide users with the features of a fully-fledged pipeline manager and an ability to execute CWL workflows anywhere Airflow can run—from a laptop to cluster or cloud environment.*

Availability: *CWL-Airflow is available under Apache license v.2 and can be downloaded from https://barski-lab.github.io/cwl-airflow.*

Keywords: Common workflow language, workflow manager, pipeline manager, Airflow, reproducible data analysis. Workflow portability

Background

Modern biomedical research has seen a remarkable increase in the production and computational analysis of large datasets, leading to an urgent need to share standardized analytical techniques. However, of the more than one hundred computational workflow systems used in biomedical research, most define their own specifications for computational pipelines [1], (https://github.com/pditommaso/awesome-pipeline). Furthermore, the evolving complexity of computational tools and pipelines makes it nearly impossible to reproduce computationally heavy studies or to repurpose published analytical workflows. Even when the tools are published, the lack of a precise description of the operating system environment and component software versions can lead to inaccurate reproduction of the analyses—or analyses failing altogether when executed in a different environment. To ameliorate this situation, a team of researchers and software developers formed the Common Workflow Language (CWL) working group (http://www.commonwl.org/) with the intent of establishing a specification for describing analysis workflows and tools in a way that makes them portable and scalable across a variety of software and hardware environments. CWL specification provides a set of formalized rules that can be used to describe each command line tool and its parameters, and optionally a container (e.g., a Docker or Singularity[2] image) with the tool already installed. CWL workflows are composed of one or more of such command line tools. Thus, CWL provides a description of the working environment and version of each tool, how the tools are "connected" together, and what parameters were used in the pipeline. Researchers using CWL are then able to deposit descriptions of their tools and workflows into a repository (e.g., dockstore.org) upon publication, thus making their analyses reusable by others.

After version 1.0 of the CWL standard [3] and the reference executor, cwl-tool, were finalized in 2016, developers began adapting the existing pipeline managers to use CWL. For example, companies such as Seven Bridges Genomics and Curoverse are developing the commercial platforms Rabix [4] and Arvados (https://arvados.org) whereas academic developers (*e.g.*, Galaxy [5], Toil [6] and others) are adding CWL support to their pipeline managers (See Table S1 for the comparison of their features).

Airflow (http://airflow.incubator.apache.org) is a lightweight workflow manager initially developed by AirBnB, which is currently an Apache Incubator project, and is available under a permissive Apache license. Airflow executes each workflow as a Directed Acyclic Graph (DAG) of tasks, in which tasks comprising the workflow are organized in a way that reflects their relationships and dependencies. DAG objects are initiated from Python scripts placed in a designated folder. Airflow has a modular architecture and can distribute tasks to an arbitrary number of workers, possibly across multiple servers, while adhering to the task sequence and dependencies specified in the DAG. Unlike many of the more complicated platforms, Airflow imposes little overhead, is easy to install, and can be used to run task-based workflows in various environments ranging from standalone desktops and servers to Amazon or Google cloud platforms. It also scales horizontally on clusters managed by Apache Mesos [7] and may be configured to send tasks to Celery (http://www.celeryproject.org) task queue. Here we present an extension of Airflow, allowing it to run CWL-based pipelines. This gives us a lightweight workflow management system with full support for CWL, the most promising scientific workflow description language.

Methods

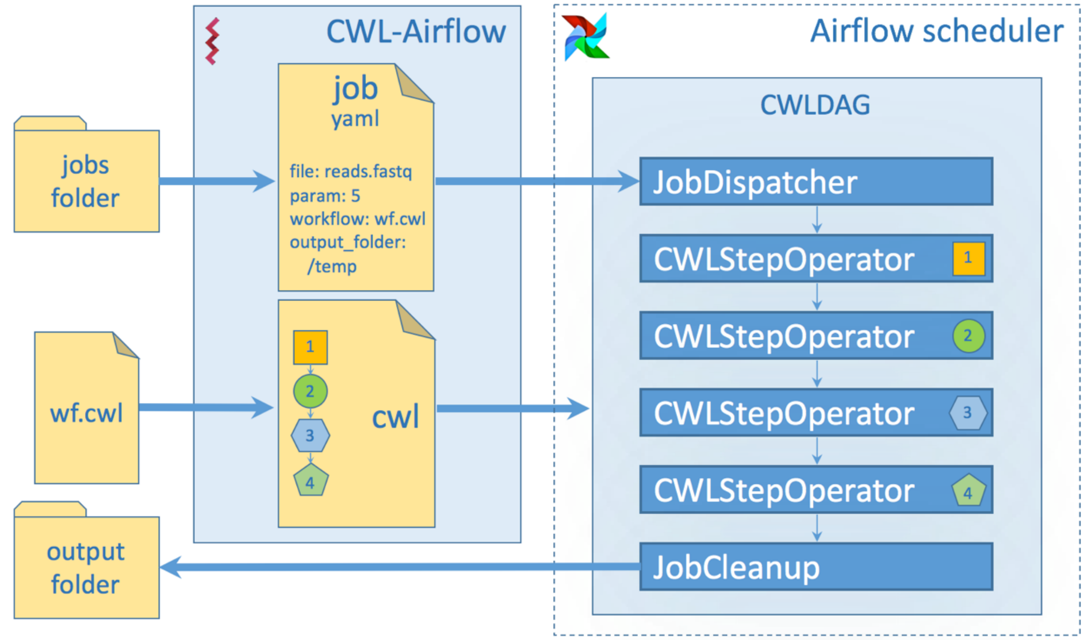

Figure 1. CWL-Airflow structural scheme

CWL-Airflow package extends Airflow's functionality with the ability to parse and execute workflows written with the current CWL specification (v1.0 [3]). The Apache Airflow code is extended with a Python package that defines four basic classes—*CWLStepOperator*, *JobDispatcher*, *JobCleanup*, and *CWLDAG*. Additionally, the automatically generated *cwl_dag.py* script is placed in the DAGs folder. The *cwl_dag.py* script is used by the Airflow scheduler to generate DAGs on the basis of job parameters and corresponding CWL workflow descriptor files (Fig. 1).

Airflow scheduler periodically runs *cwl_dag.py* which monitors job folder (set in the Airflow configuration file) for new jobs. To run a CWL workflow, a file describing the job is placed in the *jobs* folder (Fig. 1). The job file includes workflow specific input parameters (e.g. input file locations and other parameters) and three mandatory fields: *workflow* (absolute path to the CWL descriptor file to be run with this job), *output_folder* (absolute path to the folder where all the output files should be moved after successful pipeline execution) and *uid* (unique identifier for the run). The *cwl_dag.py* script parses the job file from the *jobs* folder and creates CWLDAG class instances using the job files and corresponding CWL workflows.

CWLDAG is a collection of *CWLStepOperator* tasks, which are organized in a graph that reflects the workflow steps and their relationships and dependencies. Additionally, *JobDispatcher* and *JobCleanup* tasks are added to the CWLDAG. *JobDisptacher* is used to serialize the input parameters job file and provide the CWLDAG with input data; *JobCleanup* returns the calculated results to the output folder. CWLDAG is used by Airflow to run a workflow with a structure identical to the original CWL descriptor file.

Results

ChIP-Seq analysis with CWL-Airflow

As an example, we used a workflow for basic analysis of ChIP-Seq data (Fig. 2). This workflow is a CWL version of a Python pipeline from BioWardrobe [8,9]. It starts by using BowTie [10] to perform alignment to a reference genome, resulting in an unsorted SAM file. The

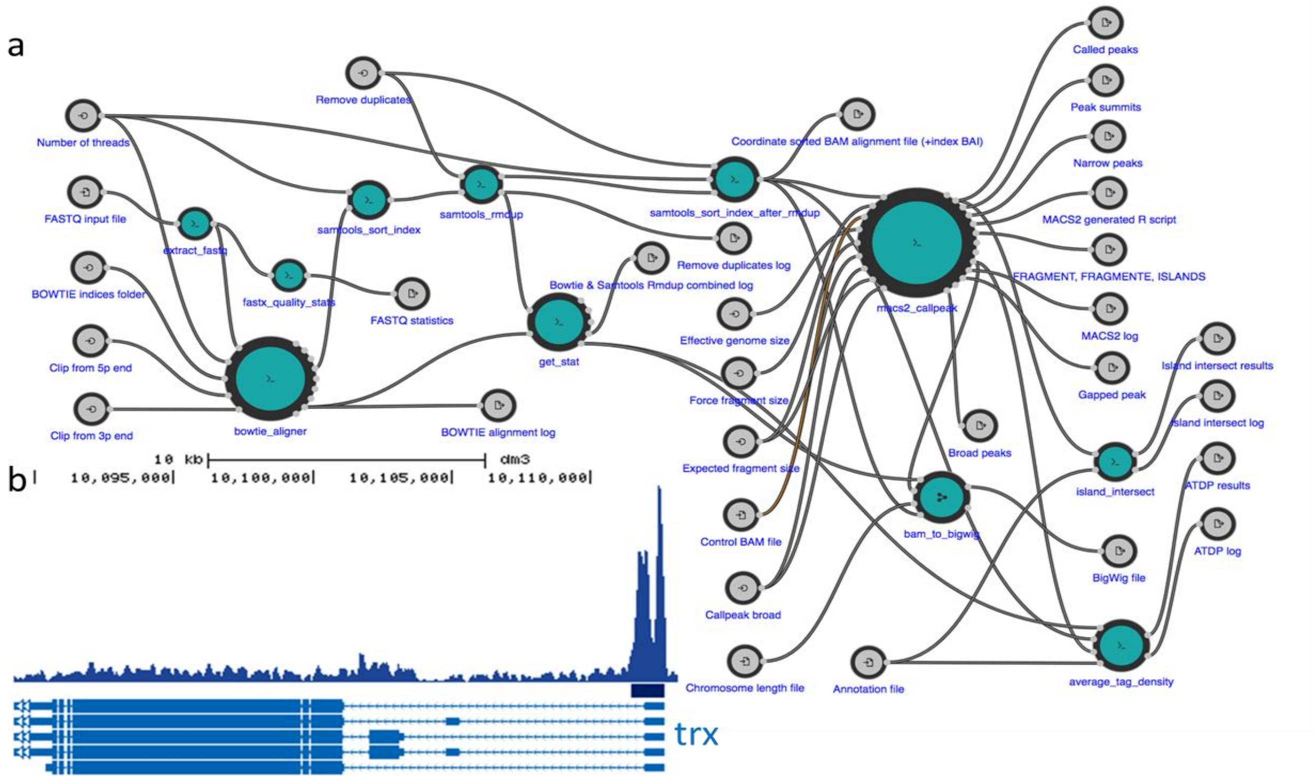

Figure 2. Using CWL-Airflow for analysis of ChIP-Seq data. (a) ChIP-Seq data analysis pipeline visualized by Rabix Composer. (b) Drosophila embryo H3K4me3 ChIP-Seq data (SRR1198790) were processed by our pipeline and CWL-Airflow. UCSC genome browser view of tag density and peaks at trx gene is shown.

SAM file is then sorted and indexed with SAMtools [11] to obtain a BAM file and a BAI index. Next MACS2 [12] is used to call peaks and to estimate fragment size. In the last few steps, the coverage by estimated fragments is calculated from the BAM file and is reported in bigWig format (Fig. 2). The pipeline also reports statistics, such as read quality, peak number and base frequency, and other troubleshooting information using tools such as FASTX-Toolkit (http://hannonlab.cshl.edu/fastx_toolkit/) and BamTools (https://github.com/pezmaster31/bamtools). The directions how to run sample pipeline can be found at https://barski-lab.github.io/cwl-airflow/#running-sample-chip-seq-se-workflow. Execution time in CWL-Airflow was similar to that reference implementation (Table 1).

CWL-Airflow package includes two additional demo workflows: (i) identification of super-enhancers [13] and (ii) a simplified version of Xenbase [14] RNA-Seq pipeline. More pipelines can be found elsewhere. In particular, BioWardrobe’s [8] pipelines for analysis of single

and paired-end ChIP-Seq, stranded and un-stranded, single and paired RNA-Seq are available on GitHub (https://github.com/Barski-lab/workflows). Additional collections of tools are available in Rabix Composer, a graphical CWL Editor from Seven Bridges and at the dockstore (http://dockstore.org).

Portability of CWL analysis

The key promise of CWL is the portability of analysis. Portability refers to the ability to seamlessly run a containerized CWL pipeline developed for one CWL platform on another CWL platform allowing users to easily share computational workflows. To check whether CWL-Airflow can use pipelines developed by others, we downloaded an alternative workflow for analysis of ChIP-Seq data developed by ENCODE Data Coordination Center[15][16] using a test dataset (CEBPB ChIP-Seq in A549 cells, ENCODE accession: ENCSR000DYI). CWL-Airflow was able to run the pipeline and produced results identical to those obtained with the reference cwl-tool. Execution time is shown in Table 1. These results confirm that CWL-Airflow complies with CWL specifications, support portability and can perform analysis in a reproducible manner. Additional testing of pipeline portability is currently conducted as a part of GA4GH workflow portability challenge[17].

Table 1. CWL-Airflow and cwltool execution time (seconds ± SEM, n=3).

	CWL-Airflow	cwltool
BioWardrobe ChIP-Seq Workflow	1023±7	1035±15
ENCODE ChIP-Seq Mapping Workflow	2798±31	2772±92

Discussion

CWL-Airflow is one of the first pipeline managers supporting version 1.0 of the CWL standard and provides a robust and user-friendly interface for executing CWL pipelines. Unlike more complicated pipeline managers, the installation of Airflow and the CWL-Airflow extension can be performed with a single *pip install* command. The comparison of CWL-Airflow and several other popular workflow managers is available in Table 2. Furthermore, as one of the most lightweight pipeline managers, Airflow contributes only a small amount of overhead to the overall execution of a computational pipeline (Table 1). We believe, however, that this is a small price to pay for the ability to monitor and restart task execution afforded by Airflow and better reproducibility and portability of biomedical analyses as afforded by the use of CWL. In summary, CWL-Airflow will provide users with the ability to execute CWL workflows anywhere Airflow can run—from a laptop to cluster or cloud environment.

Table 2. Comparison of selected workflow managers and engines with existing or planned support for CWL.

Feature	CWL-Airflow	Rabix Bunny	Galaxy	Toil	Arvados	Cromwell
System type	Workflow management system	Workflow runner	Workflow management system	Workflow management system	Workflow management system	Workflow management system
Programming language	Python	Java	Python	Python	Ruby	Java
License type	Apache License 2.0	Apache License 2.0	Academic Free License 3.0	Apache License 2.0	Apache License 2.0	BSD 3-Clause
Supported workflow description specifications	CWL v1.0, python based code	CWL v1.0	own XML-based language, CWL is planned	CWL v1.0, python based code	CWL v1.0, own JSON-based language	WDL, CWL is planned
DB backend	in-memory DB, MySQL, PostgreSQL, Microsoft SQL, any supported by SQLAlchemy	in-memory DB	in-memory DB, MySQL, PostgreSQL	through the job store (local directory or AWS, Azure, GCP location)	PostgreSQL	in-memory DB, MySQL
Installation	pip install	jar file	installation script, multiple steps	pip install	multiple steps, 5 nodes required	jar file
Mesos	Yes	No	Yes	Yes	No	Yes
GCP	Yes	No	Yes	Yes	Yes	Yes
AWS	Yes	No	Yes	Yes	Yes	Yes
Azure	Yes	No	No	Yes	Yes	No
OpenStack	Yes	No	No	Yes	Yes	No
HPC/HTC	Yes	No	Yes	Yes	Yes	Yes
Web interface for task monitoring and management	Yes	No	Yes	No	Yes	No
DAG tree/graph view	Yes	No	Yes	No	Yes	No
Gantt chart for task duration and overlap	Yes	No	No	No	No	No
GUI control of DAG/task execution flow	Yes	No	Yes	No	Yes	No
Rest API	Yes	No	Yes	No	Yes	Yes
Workflow execution	Yes	No	No	No	No	No

calendar scheduling						
Workflow recurring execution	Yes	No	No	No	No	No
Workflow execution timeout	Yes	No	No	Yes	No	No
Automatic task rerun if failed	Yes	No	No	Yes	Yes (in case of a temporary failure)	No
Cross DAGs task dependencies	Yes	No	No	No	No	No
Task parallelization within DAG	Yes	Yes	Yes	Yes	Yes	Yes
Dynamic DAG creation	Yes	No	No	Yes	No	No
Backfilling DAGs	Yes	No	No	No	No	No
Tasks prioritizations	Yes	No	No	No	No	No
Workflow portability due to standardized and commonly used workflow description specification	Yes	Yes	No	Yes	Yes	No
Simultaneous running multiple workflows	Yes	No	Yes	Yes	Yes	Yes
Implements workflow queue	Yes	No	Yes	Yes	Yes	Yes

Abbreviations:

CWL: Common workflow language

DAG: Directed Acyclic Graph

ChIP-Seq: Chromatin ImmunoPrecipitation - Sequencing

Declarations

Ethics approval and consent to participate: Not applicable.

Consent to publish: Not applicable.

Availability of data and materials: Now new datasets or materials were generated. The source code is available under Apache license v.2 and can be downloaded from https://barski-lab.github.io/cwl-airflow.

Competing Interests: AVK and AB are co-founders of Datirium, LLC. Datirium, LLC provides bioinformatics software support services.

Funding: The project was supported in part by Center for Clinical & Translational Research and Training (NIH CTSA grant UL1TR001425) and by NIH NIGMS New Innovator Award to AB (DP2GM119134). The funders had no role in study design, data collection and analysis, decision to publish, or preparation of the manuscript.

Author contributions statement: AVK and AB conceived the project, AVK and MK wrote the software, MK, AVK and AB wrote and reviewed the manuscript.

Acknowledgements: The authors thank all members of the CWL working group for their support and Shawna Hottinger for editorial assistance.

References

1. Leipzig J. A review of bioinformatic pipeline frameworks. Brief Bioinform. 2017;18:530–6.

2. Kurtzer GM, Sochat V, Bauer MW. Singularity: Scientific containers for mobility of compute. PLoS One. 2017;12.

3. Amstutz P, Crusoe MR, Tijanic N, Chapman B, Chilton J, Heuer M, et al. Common Workflow Language, v1.0 [Internet]. 2016 [cited 2017 Jul 17]. Available from: https://figshare.com/articles/Common_Workflow_Language_draft_3/3115156

4. Kaushik G, Ivkovic S, Simonovic J, Tijanic N, Davis-Dusenbery B, Kural D. RABIX: an open-source workflow executor supporting recomputability and interoperability of workflow descriptions. Pac Symp Biocomput. 2016;22:154–65.

5. Giardine B, Riemer C, Hardison RC, Burhans R, Elnitski L, Shah P, et al. Galaxy: a platform for interactive large-scale genome analysis. Genome Res. 2005;15:1451–5.

6. Vivian J, Rao A, Nothaft FA, Ketchum C, Armstrong J, Novak A, et al. Rapid and efficient analysis of 20,000 RNA-seq samples with Toil. bioRxiv. 2016;2:062497.

7. Hindman B, Konwinski A, Zaharia M, Ghodsi A, Joseph AD, Katz R, et al. Mesos: A platform for fine-grained resource sharing in the data center. Proc 8th USENIX Conf Networked Syst Des Implement. 2011;295.

8. Kartashov A V, Barski A. BioWardrobe: an integrated platform for analysis of epigenomics and transcriptomics data. Genome Biol. 2015;16:158.

9. Vallabh S, Kartashov A V., Barski A. Analysis of ChIP-Seq and RNA-Seq Data with BioWardrobe. Methods Mol Biol. 2018;1783:343–60.

10. Langmead B, Trapnell C, Pop M, Salzberg SL. Ultrafast and memory-efficient alignment of short DNA sequences to the human genome. Genome Biol. 2009;10:R25.

11. Li H, Handsaker B, Wysoker A, Fennell T, Ruan J, Homer N, et al. The Sequence Alignment/Map format and SAMtools. Bioinformatics. 2009;25:2078–9.

12. Zhang Y, Liu T, Meyer CA, Eeckhoute J, Johnson DS, Bernstein BE, et al. Model-based analysis of ChIP-Seq (MACS). Genome Biol. 2008/09/19. 2008;9:R137.

13. Hnisz D, Abraham BJ, Lee TI, Lau A, Saint-André V, Sigova A a, et al. Super-enhancers in the control of cell identity and disease. Cell. 2013;155:934–47.

14. Karimi K, Fortriede JD, Lotay VS, Burns KA, Wang DZ, Fisher ME, et al. Xenbase: a genomic, epigenomic and transcriptomic model organism database. Nucleic Acids Res. 2018;46:D861–8.

15. Landt SG, Marinov GK, Kundaje A, Kheradpour P, Pauli F, Batzoglou S, et al. ChIP-seq guidelines and practices of the ENCODE and modENCODE consortia. Genome Res. 2012;22:1813–31.

16. ENCODE ChIP-Seq pipeline [Internet]. Available from: https://github.com/ENCODE-DCC/pipeline-container

17. GA4GH-DREAM Workflow Execution Challenge [Internet]. Available from: https://www.synapse.org/#!Synapse:syn8507133/wiki/415976

Personal Cover
Click here to download Personal Cover cover2giga.docx

Cincinnati Children's
changing the outcome together

UNIVERSITY OF Cincinnati

Allergy & Immunology

Marc E. Rothenberg, MD, PhD
Professor
Director, Division of Allergy & Immunology
Director, Cincinnati Center for Eosinophilic Disorders
Phone 513-803-0257, Fax 513-636-3310
rothenberg@cchmc.org

Amal H. Assa'ad, MD
Professor
Associate Director, Division of Allergy & Immunology
Director, Allergy & Immunology Clinical Services
Phone 513-636-7944, Fax 513-636-5835
amal.assa'ad@cchmc.org

Simon P. Hogan, PhD
Associate Professor
Director, Allergy & Immunology Research
Phone 513-636-6620, Fax 513-636-3310
simon.hogan@cchmc.org

Kimberly A. Risma, MD, PhD
Associate Professor
Director, Allergy & Immunology Fellowship Program
Phone 513-803-4230, Fax 513-636-3310
kimberly.risma@cchmc.org

J. Pablo Abonia, MD
Associate Professor

Artem Barski, PhD
Associate Professor

Sandy Durrani, MD
Assistant Professor

Thomas J. Fischer, MD
Adjunct Professor

Patricia C. Fulkerson, MD, PhD
Assistant Professor

Michelle B. Lierl, MD
Staff Physician

Andrew W. Lindsley, MD, PhD
Instructor

Stephanie L. Logsdon, MD
Assistant Professor

Ting Wen, PhD
Instructor

Nives Zimmermann, MD
Associate Professor

Kevin Titus, MBA
Business Director

Melissa Mingler, MS, MBA
Business Manager

February 12, 2019

Editor,

GigaScience

Oxford University Press

Dear Editor,

We are submitting our manuscript titled "CWL-Airflow: a lightweight pipeline manager supporting Common Workflow Language" for your consideration for publication in GigaScience as a "Technology Note". Given the GigaScience's emphasis on reproducibility in data analysis, we believe our paper will be a strong fit for your journal.

Massive growth in the amount of research data and computational analysis has led to increased utilization of pipeline managers in biomedical computational research. However, each of more than 100 such managers uses its own way to describe pipelines, leading to difficulty porting workflows to different environments and therefore poor reproducibility of computational studies. For this reason, the Common Workflow Language (CWL) was recently introduced as a specification for platform-independent workflow description, and work began to transition existing pipelines and workflow managers to CWL.

In this paper, we describe one of the first CWL-capable workflow managers and a CWL pipeline for the analysis of ChIP-Seq data. CWL-Airflow is an extension for the Apache Airflow pipeline manager (initially developed by AirBnB). CWL-Airflow utilizes the most recent CWL v1.02 specification and can be used to run workflows on standalone MacOS/Linux servers, on clusters, or on a variety of cloud platforms. Unlike some of the other platforms in development (e.g., Arvados and Seven Bridges), CWL-Airflow is easy to install and does not consume excessive resources, making it ideal for managing data analysis in research laboratories. A sample CWL pipeline for processing of ChIP-Seq data is provided. Although, we are not including specific biological results in the manuscript, we believe that the great need for reproducibility and portability of computational analysis justifies publication of our paper in your journal.

Given the strong interest in CWL among computational biologists, we hope that our manuscript and CWL-Airflow will be of high interest to the readership of GigaScience.

Sincerely yours,

Artem Barski,
Associate Professor,
Divisions of Allergy & Immunology and Human Genetics
Director, Epigenomics Data Analysis Core

Cincinnati Children's Hospital Medical Center
University of Cincinnati College of Medicine

S.6.409 MLC7028,
240 Albert Sabin Way,
Cincinnati, OH 45229-3039
Phone: (513) 636-1851
Fax: (513) 636-3310
artem.barski@cchmc.org
