# Supplementary material for: CWL-Airflow: a lightweight pipeline manager supporting Common Workflow Language
Source: Gigascience. 2019 Jul 18;8(7):giz084. doi: 10.1093/gigascience/giz084 (PMC6639121; doi:10.1093/gigascience/giz084)
Supplement: giz084_GIGA-D-19-00044_Revision_1 [file giz084_giga-d-19-00044_revision_1.pdf]

GigaScience

CWL-Airflow: a lightweight pipeline manager supporting Common Workflow Language

--Manuscript Draft--

**Manuscript Number:**	GIGA-D-19-00044R1
**Full Title:**	CWL-Airflow: a lightweight pipeline manager supporting Common Workflow Language
**Article Type:**	Technical Note
**Funding Information:**	National Institute of General Medical Sciences (DP2GM119134) — Dr Artem Barski
**Abstract:**	Background: Massive growth in the amount of research data and computational analysis has led to increased utilization of pipeline managers in biomedical computational research. However, each of more than 100 such managers uses its own way to describe pipelines, leading to difficulty porting workflows to different environments and therefore poor reproducibility of computational studies. For this reason, the Common Workflow Language (CWL) was recently introduced as a specification for platform-independent workflow description, and work began to transition existing pipelines and workflow managers to CWL.<br>Findings: Herein, we present CWL-Airflow, an package that adds support for CWL to the Apache Airflow pipeline manager. CWL-Airflow utilizes CWL version 1.0 (v1.0) specification and can run workflows on standalone MacOS/Linux servers, on clusters, or on a variety of cloud platforms. A sample CWL pipeline for processing of chromatin immunoprecipitation sequencing (ChIP-Seq) data is provided.<br>Conclusions: CWL-Airflow will provide users with the features of a fully-fledged pipeline manager and an ability to execute CWL workflows anywhere Airflow can run—from a laptop to a cluster or cloud environment.<br>Availability: CWL-Airflow is available under Apache License, Version 2.0 (Apache-2.0) and can be downloaded from https://barski-lab.github.io/cwl-airflow, http://doi.org/10.5281/zenodo.2852870, RRID: SCR_017196.
**Corresponding Author:**	Artem Barski<br>Cincinnati Children's Hospital Medical Center<br>UNITED STATES
**Corresponding Author Secondary Information:**	
**Corresponding Author's Institution:**	Cincinnati Children's Hospital Medical Center
**Corresponding Author's Secondary Institution:**	
**First Author:**	Michael Kotliar, MS
**First Author Secondary Information:**	
**Order of Authors:**	Michael Kotliar, MS
	Andrey V. Kartashov, MS
	Artem Barski, PhD
**Order of Authors Secondary Information:**	
**Response to Reviewers:**	See the file attached as Supplementary Material.
**Additional Information:**	
**Question**	**Response**
Are you submitting this manuscript to a special series or article collection?	No
**Experimental design and statistics**	Yes

Powered by Editorial Manager® and ProduXion Manager® from Aries Systems Corporation

Full details of the experimental design and statistical methods used should be given in the Methods section, as detailed in our Minimum Standards Reporting Checklist. Information essential to interpreting the data presented should be made available in the figure legends.<br><br>Have you included all the information requested in your manuscript?	
**Resources**<br><br>A description of all resources used, including antibodies, cell lines, animals and software tools, with enough information to allow them to be uniquely identified, should be included in the Methods section. Authors are strongly encouraged to cite Research Resource Identifiers (RRIDs) for antibodies, model organisms and tools, where possible.<br><br>Have you included the information requested as detailed in our Minimum Standards Reporting Checklist?	Yes
**Availability of data and materials**<br><br>All datasets and code on which the conclusions of the paper rely must be either included in your submission or deposited in publicly available repositories (where available and ethically appropriate), referencing such data using a unique identifier in the references and in the "Availability of Data and Materials" section of your manuscript.<br><br>Have you have met the above requirement as detailed in our Minimum Standards Reporting Checklist?	Yes

Manuscript

Click here to view linked References

Click here to access/download;Manuscript;cwl_airflow-May20.docx

CWL-Airflow: a lightweight pipeline manager supporting Common Workflow Language

Michael Kotliar[1,*], Andrey V. Kartashov[1,*], and Artem Barski[1,2,#]

[1] Division of Allergy and Immunology, [2] Division of Human Genetics, Cincinnati Children's Hospital Medical Center and Department of Pediatrics, College of Medicine, University of Cincinnati, Cincinnati, OH

* Joint first author; [#] To whom correspondence should be addressed: Artem.Barski@cchmc.org.

Emails:

MK: michael.kotliar@cchmc.org

AVK: andrey.kartashov@cchmc.org

AB: artem.barski@cchmc.org

Short title: CWL-Airflow pipeline manager

Abstract

Background: Massive growth in the amount of research data and computational analysis has led to increased utilization of pipeline managers in biomedical computational research. However, each of more than 100 such managers uses its own way to describe pipelines, leading to difficulty porting workflows to different environments and therefore poor reproducibility of computational studies. For this reason, the Common Workflow Language (CWL) was recently introduced as a specification for platform-independent workflow description, and work began to transition existing pipelines and workflow managers to CWL.

Findings: Herein, we present CWL-Airflow, an package that adds support for CWL to the Apache Airflow pipeline manager. CWL-Airflow utilizes CWL version 1.0 (v1.0) specification and can run workflows on standalone MacOS/Linux servers, on clusters, or on a variety of cloud

platforms. A sample CWL pipeline for processing of chromatin immunoprecipitation sequencing (ChIP-Seq) data is provided.

Conclusions: CWL-Airflow will provide users with the features of a fully-fledged pipeline manager and an ability to execute CWL workflows anywhere Airflow can run—from a laptop to a cluster or cloud environment.

Availability: CWL-Airflow is available under Apache License, Version 2.0 (Apache-2.0) and can be downloaded from *https://barski-lab.github.io/cwl-airflow*, *http://doi.org/10.5281/zenodo.2852870*, RRID: SCR_017196.

Keywords: Common workflow language, workflow manager, pipeline manager, Airflow, reproducible data analysis, workflow portability

Background

Modern biomedical research has seen a remarkable increase in the production and computational analysis of large datasets, leading to an urgent need to share standardized analytical techniques. However, of the more than one hundred computational workflow systems used in biomedical research, most define their own specifications for computational pipelines [1,2]. Furthermore, the evolving complexity of computational tools and pipelines makes it nearly impossible to reproduce computationally heavy studies or to repurpose published analytical workflows. Even when the tools are published, the lack of a precise description of the operating system environment and component software versions can lead to inaccurate reproduction of the analyses—or analyses failing altogether when executed in a different environment. To ameliorate this situation, a team of researchers and software developers formed the Common Workflow Language (CWL) working group [3] with the intent of establishing a specification for describing

analysis workflows and tools in a way that makes them portable and scalable across a variety of software and hardware environments. The CWL specification provides a set of formalized rules that can be used to describe each command line tool and its parameters, and optionally a container (e.g., a Docker [4] or Singularity [5] image) with the tool already installed. CWL workflows are composed of one or more of such command line tools. Thus, CWL provides a description of the working environment and version of each tool, how the tools are "connected" together, and what parameters were used in the pipeline. Researchers using CWL are then able to deposit descriptions of their tools and workflows into a repository (e.g., dockstore.org) upon publication, thus making their analyses reusable by others.

After version 1.0 of the CWL standard [6] and the reference executor, cwltool, were finalized in 2016, developers began adapting the existing pipeline managers to use CWL. For example, companies such as Seven Bridges Genomics, Inc. and Curoverse, Inc. are developing the commercial platforms Rabix [7] and Arvados [8], whereas academic developers (e.g., Galaxy [9], Toil [10] and others) are adding CWL support to their pipeline managers (See Discussion).

Airflow [11] is a lightweight workflow manager initially developed by AirBnB, Inc., which has now graduated from Apache Incubator, and is available under a permissive Apache license. Airflow executes each workflow as a directed acyclic graph (DAG) of tasks that have directional, noncircular dependencies. The tasks are usually atomic and are not supposed to share any resources with each other; therefore, they can be run independently. The DAG describes relationships between the tasks and defines their order of execution. The DAG objects are initiated from Python scripts placed in a designated folder. Airflow has a modular architecture and can distribute tasks to an arbitrary number of workers and across multiple servers while adhering to the task sequence and dependencies specified in the DAG. Unlike many of the more complicated platforms, Airflow

imposes little overhead, is easy to install, and can be used to run task-based workflows in various environments ranging from standalone desktops and servers to Amazon or Google cloud platforms. It also scales horizontally on clusters managed by Apache Mesos [12] and may be configured to send tasks to the Celery [13] task queue. Herein, we present an extension of Airflow, allowing it to run CWL-based pipelines. Altogether, this gives us a lightweight workflow management system with full support for CWL, the most promising scientific workflow description language.

Methods

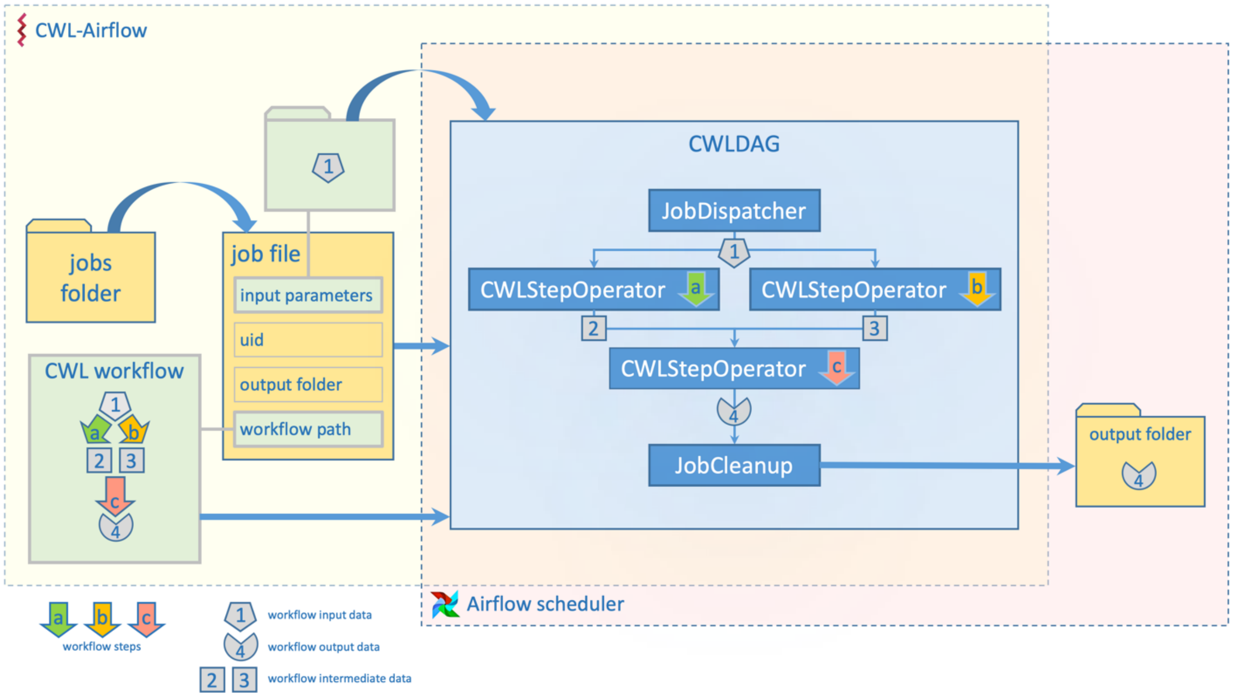

Figure 1. *CWL-Airflow diagram. The job file contains information about the CWL workflow and inputs. CWL-Airflow creates a CWLDAG-class instance on the basis of the workflow structure and executes it in Airflow. The results are saved to the output folder.*

The CWL-Airflow package extends Airflow's functionality with the ability to parse and execute workflows written with the CWL version 1.0 (v1.0) specification [6]. CWL-Airflow can be easily integrated into the Airflow scheduler logic as shown in the structure diagram in Figure

1. The Apache Airflow code is extended with a Python package that defines four basic classes—*JobDispatcher*, *CWLStepOperator*, *JobCleanup* and *CWLDAG*. Additionally, the automatically generated *cwl_dag.py* script is placed in the DAGs folder. While periodically loading DAGs from the DAGs folder, the Airflow scheduler runs the *cwl_dag.py* script and creates DAGs on the basis of the available jobs and corresponding CWL workflow descriptor files.

In order to run a CWL workflow in Airflow, a file describing the job should be placed in the *jobs* folder (Fig. 1). The jobs are described by a file in JSON or YAML format that includes workflow-specific *input parameters* (e.g., input file locations) and three mandatory fields: *workflow* (absolute path to the CWL descriptor file to be run with this job), *output_folder* (absolute path to the folder where all the output files should be moved after successful pipeline execution) and *uid* (unique identifier for the run). CWL-Airflow parses every job file from the *jobs* folder, loads corresponding CWL workflow descriptor file and creates a CWLDAG-class instance on the basis of the workflow structure and *input parameters* provided in the job file. The *uid* field from the job file is used to identify the newly created CWLDAG-class instance.

CWLDAG is a class for combining the tasks into a DAG that reflecta the CWL workflow structure. Every *CWLStepOperator* task corresponds to a workflow step and depends on others on the basis of the workflow step inputs and outputs. This implements dataflow principles and architecture that are missing in Airflow. Additionally, the *JobDispatcher* and *JobCleanup* tasks are added to the DAG. *JobDisptacher* is used to serialize the *input parameters* from the job file and provide the pipeline with the input data; *JobCleanup* returns the calculated results to the *output folder*. When the Airflow scheduler executes the pipeline from the CWLDAG, it runs the workflow with the structure identical to the CWL descriptor file used to create this graph.

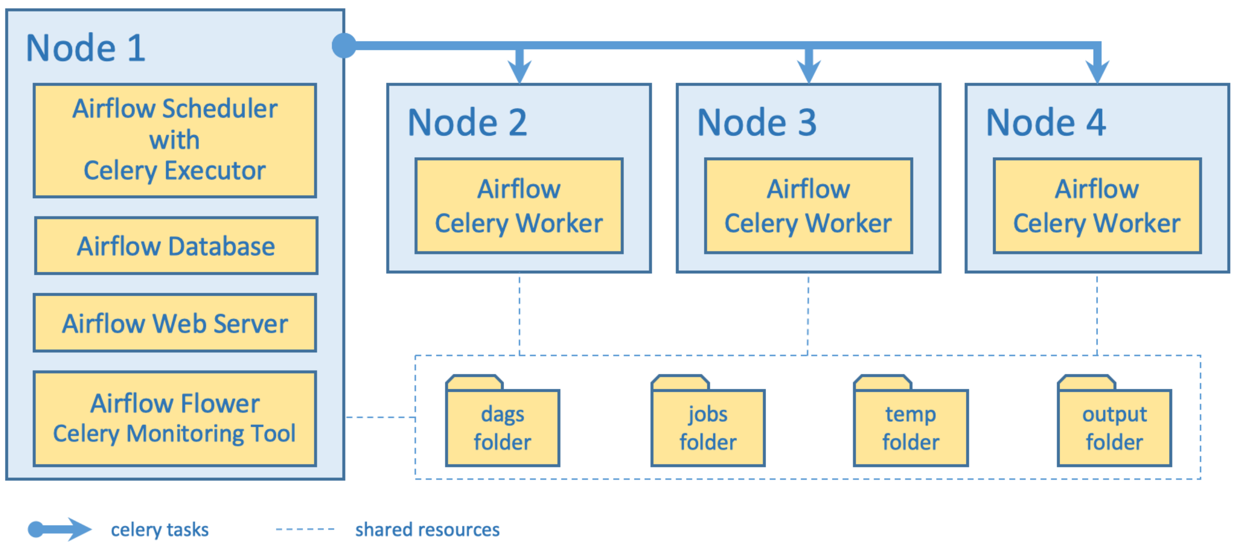

Figure 2. *Structure diagram for scaling out CWL-Airflow with a Celery cluster of 4 nodes. Node 1 runs the Airflow database to save task metadata and the Airflow scheduler with the Celery executor to submit tasks for processing to the Airflow celery workers on nodes 2, 3 and 4. The Airflow and Flower (Celery) web servers allow for monitoring and controlling of the task execution process. All nodes have shared access to the dags, jobs, temp and output folders.*

Though running CWL-Airflow on a single node may be sufficient in most of the cases, it is worth switching to the multi-node configuration (Fig. 2) for computationally intensive pipelines. Airflow uses the Celery task queue to distribute processing over the multiple nodes. Celery provides the mechanisms for queueing and assigning tasks to the multiple workers, whereas the Airflow scheduler uses Celery executor to submit tasks to the queue. The Celery system helps to not only balance the load over the different machines, but also define task priorities by assigning them to the separate queues.

The example of a CWL-Airflow Celery cluster of 4 nodes is shown in Figure 2. The tasks are submitted to the queue by the node 1 and executed by either of the 3 workers (nodes 2, 3 and 4). Node 1 runs two mandatory components—the Airflow database and scheduler. The latter

schedules the task execution by adding tasks to the queue. All Celery workers are subscribed to the same task queue. Whenever an arbitrary worker pulls a new task from the queue, it runs the task and returns the execution results. For the sequential steps, the Airflow scheduler submits the next tasks to the queue. During the task execution, intermediate data are kept in the *temp* folder. Upon successful pipeline completion, all output files are moved to the *output* folder. Both the *temp* and *output* folders, as well as the *dags* and *jobs* folders, are shared among all the nodes of the cluster. Optionally, node 1 can also run the Airflow webserver (Fig. 3) and the Celery monitoring tool Flower (Fig. 4) to provide users with the pipeline execution details.

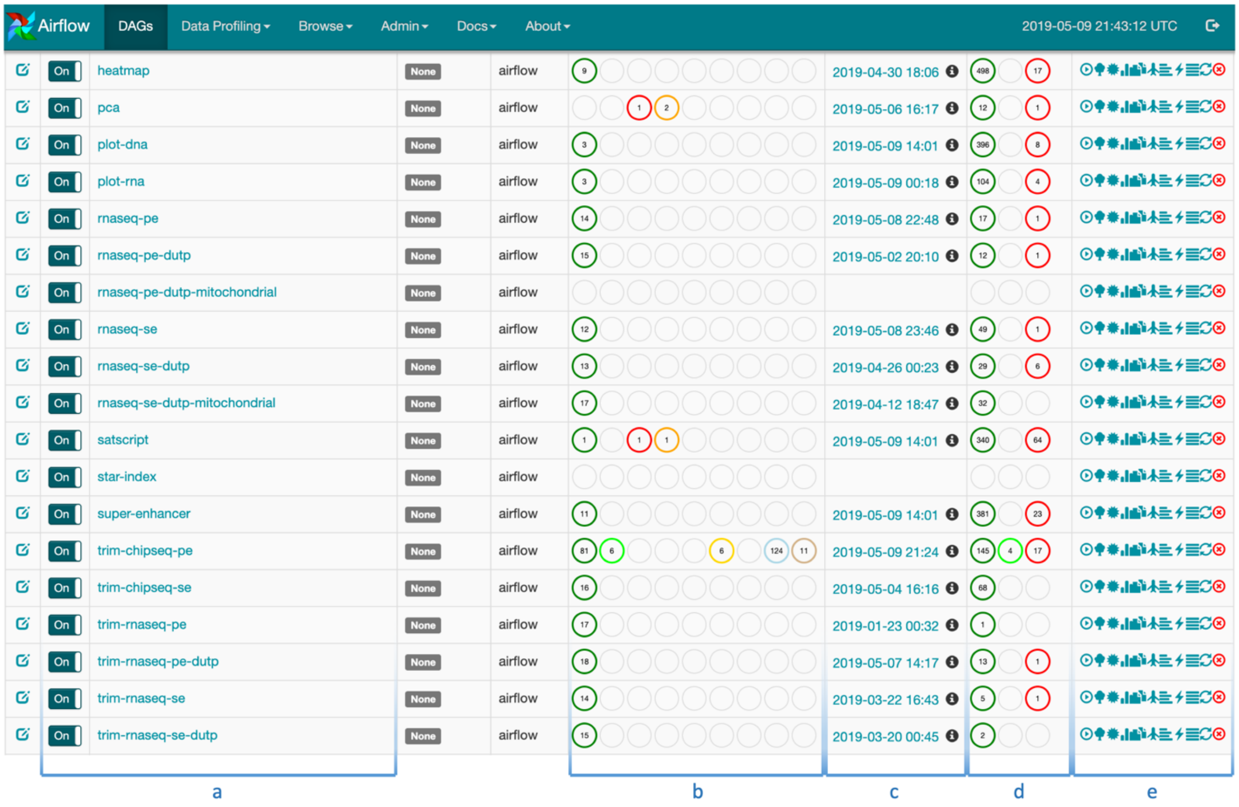

Figure 3. *Airflow web interface. The DAGs tab shows the list of the available pipelines (a) and their latest execution dates (c) and number of active, succeeded and failed runs (d) and workflow step statuses (b). The buttons on the right (e) allow a user to control pipeline execution and obtain additional information on the current workflow and its steps.*

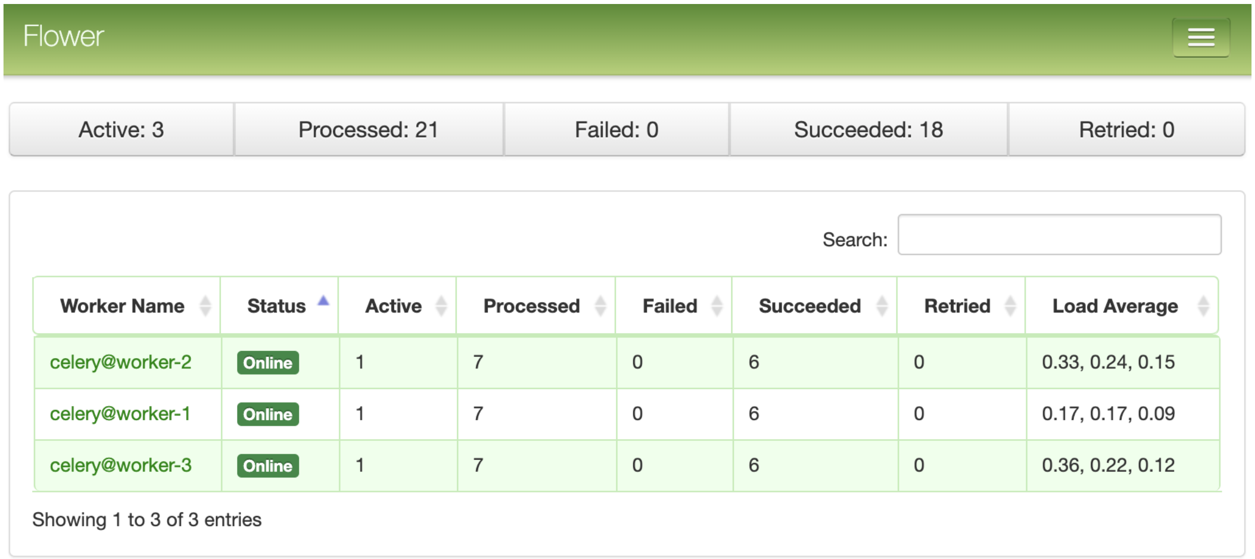

Figure 4. *Dashboard of the Celery monitoring tool Flower. Shown are the three Celery workers, their current status and load information.*

Results

ChIP-Seq analysis with CWL-Airflow

As an example, we used a workflow for basic analysis of chromatin immunoprecipitation sequencing (ChIP-Seq) data [14] (Fig. 5, Research object:Additional file 1). This workflow is a CWL version of a Python pipeline from BioWardrobe [15,16]. It starts by using BowTie [17] to perform alignment to a reference genome, resulting in an unsorted SAM file. The SAM file is then sorted and indexed with SAMtools [18] to obtain a BAM file and a BAI index. Next MACS2 [19] is used to call peaks and to estimate fragment size. In the last few steps, the coverage by estimated fragments is calculated from the BAM file and is reported in bigWig format (Fig. 5). The pipeline also reports statistics, such as read quality, peak number and base frequency, and other troubleshooting information using tools such as FASTX-Toolkit [20] and BamTools [21]. The

directions for how to run a sample pipeline can be found on the CWL-Airflow webpage [14]. Execution time in CWL-Airflow was similar to that of reference implementation (Table 1).

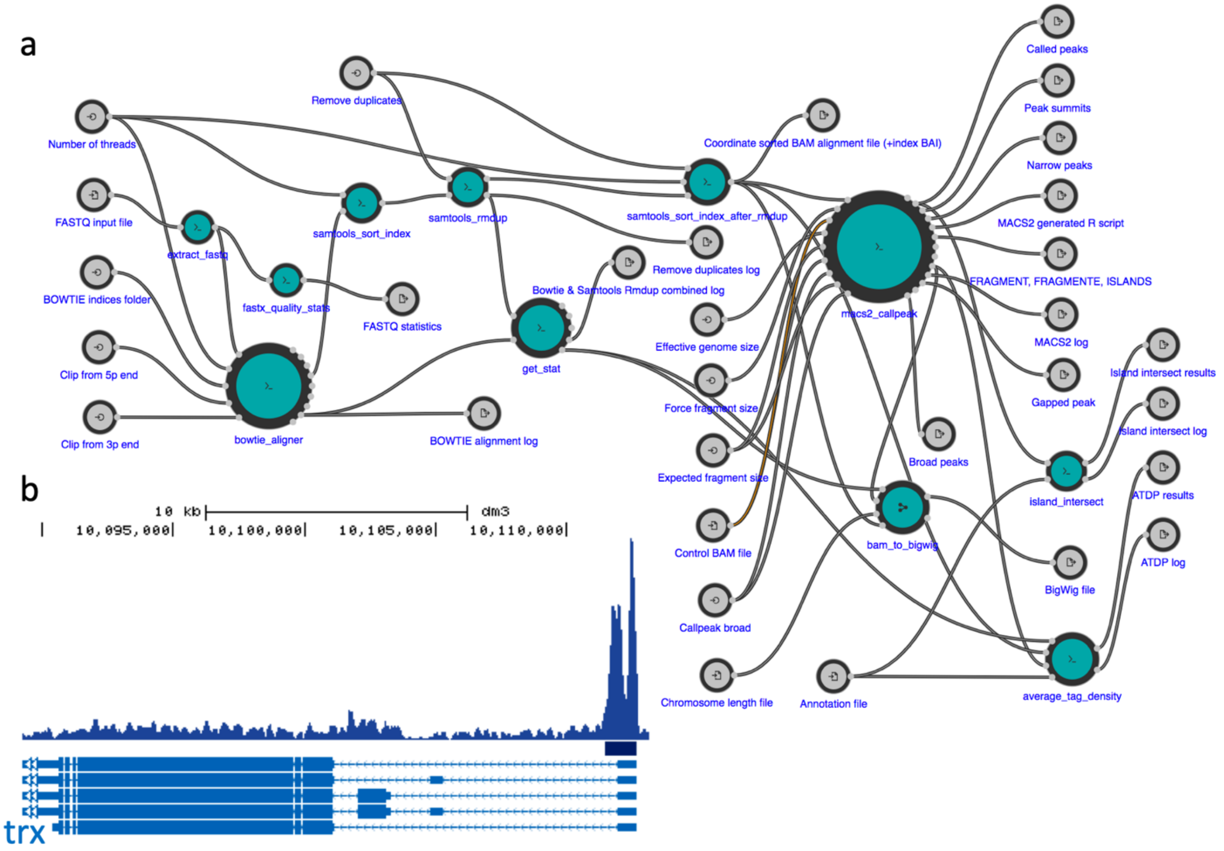

__Figure 5.__ Using CWL-Airflow for analysis of ChIP-Seq data. (a) ChIP-Seq data analysis pipeline visualized by Rabix Composer. (b) Drosophila melanogaster embryo histone 3, lysine 4 trimethylation (H3K4me3) ChIP-Seq data (SRR1198790) were processed by our pipeline and CWL-Airflow. UCSC genome browser view of tag density and peaks at the trx gene is shown.

__Table 1.__ CWL-Airflow and cwltool average execution time

Pipeline	CWL-Airflow (sec ± SEM, n = 3)		Cwltool (sec ± SEM, n = 3)
	1 node<br>1 workflow at a time	3 nodes<br>3 workflows at a time	1 node<br>1 workflow at a time
BioWardrobe ChIP-Seq Workflow	1141 ± 18	1231 ± 3	955 ± 1

ENCODE ChIP-Seq Mapping Workflow	3784 ± 10	3824 ± 28	3245 ± 7

ChIP-Seq, chromatin immunoprecipitation sequencing; CWL, common workflow language; SEM, standard error of the mean

The CWL-Airflow package includes two additional demo workflows: (i) an identification of super-enhancers [22] and (ii) a simplified version of the Xenbase [23] RNA-Seq pipeline. More pipelines can be found elsewhere. In particular, BioWardrobe's [15] pipelines for analysis of single-read and paired-end ChIP-Seq; stranded and un-stranded, single and paired RNA-Seq are available on GitHub [24]. Additional collections of tools are available in Rabix Composer [7], a graphical CWL Editor from Seven Bridges and at the Dockstore [25].

Portability of CWL analyses

The key promise of CWL is the portability of analyses. Portability refers to the ability to seamlessly run a containerized CWL pipeline developed for one CWL platform on another CWL platform, allowing users to easily share computational workflows. To check whether CWL-Airflow can use pipelines developed by others, we downloaded an alternative workflow for the analysis of ChIP-Seq data developed by the ENCODE Data Coordination Center [26,27] using a test dataset (CEBPB ChIP-Seq in A549 cells, ENCODE accession: ENCSR000DYI). CWL-Airflow was able to run the pipeline and produced results identical to those obtained with the reference cwltool. The execution time is shown in Table 1. Notably, running the tested pipelines on the single-node CWL-Airflow system increased execution time by 18%, whereas running them on the three-node CWL-Airflow cluster reduced execution time by 41% per workflow compared to the reference cwltool. These results confirm that CWL-Airflow complies with the CWL specification, supports portability and performs analysis in a reproducible manner. Additional

testing of pipeline portability is currently being conducted as a part of the Global Alliance for Genomics and Health (GA4GH) workflow portability challenge [28].

CWL-Airflow in multi-node configuration with Celery executor

To demonstrate the use of CWL-Airflow in a multi-node configuration, we set up a Celery cluster of 3 nodes with 4 CPU and 94 GB of RAM each, which each node running an instance of the Airflow Celery worker. Tasks were queued for execution by the Airflow scheduler that was launched on the first node. Communication between the Celery workers was managed by the message queueing service, RabbitMQ. RabbitMQ, as well as the Airflow database and web server, were run on the first node. Executing the two tested pipelines on the Airflow Celery cluster demonstrated only a slight slow-down on a per-run basis (Table 1).

Discussion

CWL-Airflow is one of the first pipeline managers supporting version 1.0 of the CWL standard and provides a robust and user-friendly interface for executing CWL pipelines. Unlike more complicated pipeline managers, the installation of Airflow and the CWL-Airflow extension can be performed with a single *pip install* command. Compared to the competing pipeline managers, Airflow has multiple advantages (Table 2). Specifically, Airflow provides a wide range of tools for managing the workflow execution process, such as pausing and resuming the workflow execution, stopping and restarting the individual workflow steps, restarting the workflow from a certain step and skipping part of the workflow by updating the states of the specific steps from a web-based graphical user interface (GUI). Similar to other workflow management systems, Airflow can run on clusters and the major cloud services. Unlike some of the workflow executors,

Airflow supports both Docker and Singularity containerization technologies. The latter is particularly important because many clusters do not allow the use of Docker for security reasons.

Unlike most of the other workflow managers, Airflow provides a convenient, web-based GUI that allows a user to monitor and control the pipeline execution. Within this web interface, a user can easily track the workflow execution history and collect and visualize statistics from multiple workflow runs. Similar to some of the other pipeline managers, Airflow provides a REST API (representational state transfer application program interface) that allows a user to access its functionality through the dedicated endpoints. The API can be used by other software to communicate with the Airflow system.

Airflow supports parallel workflow step execution. Step parallelization can be convenient when the workflow complexity is not high and the computational resources are not limited. However, when running multiple workflows, especially on a multi-node system, it becomes reasonable to limit parallelism and balance load over the available computing resources. Besides the standard load balancing algorithms provided by the computing environment, Airflow supports pools and queues that allow for even distribution of tasks among multiple nodes.

Addition of the CWL capability to Airflow has made it more convenient for scientific computing, in which the users are more interested in the flow of data than the tasks being executed. Though Airflow itself (and most of the pipeline managers [28]) only define workflows as sequences of steps to be executed (e.g., DAGs), the CWL description of inputs and outputs leads to better representation of data flow, which allows for a better understanding of data dependencies and produces more readable workflows.

Furthermore, as one of the most lightweight pipeline managers, Airflow contributes only a small amount of overhead to the overall execution of a computational pipeline (Table 1). We

believe that this overhead is an advantageous exchange for 1) Airflow's ability to monitor and control workflow execution and 2) CWL's enablement of better reproducibility and portability of biomedical analyses. In summary, CWL-Airflow will provide users with the ability to execute CWL workflows anywhere Airflow can run—from a laptop to a cluster or cloud environment.

Table 2. *Comparison of the open-source workflow managers and engines with existing or planned support for CWL*

Feature	Airflow & CWL-Airflow	Rabix	Toil	Cromwell	REANA	Galaxy	Arvados	CWLEXEC
Software installation complexity	single Python package	JAR<br><br>Electron application	single Python package	JAR	group of Python packages	group of Python packages<br><br>node.js application	multiple components for minimum 7 nodes system	JAR
License type	Apache License v2.0	Apache License v2.0	Apache License v2.0	Berkeley Software Distribution 3-Clause (BSD-3-Clause)	Massachusetts Institute of Technology (MIT) License	Academic Free License v3.0	Apache License v2.0<br><br>Affero General Public License (AGPL) v3.0<br><br>Creative Commons Attribution-Share-Alike (CC-BY-SA) v3.0	Apache License v2.0
Workflow description language	CWL v1.0<br><br>Python code	CWL v1.0	CWL v1.0<br><br>WDL v1.0<br><br>Python code	CWL v1.0<br><br>WDL v1.0	CWL v1.0<br><br>Serial<br><br>Yadage	XML tool file<br><br>JSON workflow file	CWL v1.0	CWL v1.0
Docker containerization	+	+	+	+	+	+	+	+
Singularity containerization	+	–	+	+	–	+	–	–
Cloud / cluster processing	+	–	+	+	+	+	+	+
Workflow execution load balancing[1]	+	–	+	+	+	+	+	+
Parallel workflow step execution	+	+	+	+	+	+	+	+

Feature	Airflow & CWL-Airflow GUI	Airflow & CWL-Airflow REST API	Airflow & CWL-Airflow CLI	Rabix GUI	Rabix REST API	Rabix CLI	Toil GUI	Toil REST API	Toil CLI	Cromwell GUI	Cromwell REST API	Cromwell CLI	REANA GUI	REANA REST API	REANA CLI	Galaxy GUI	Galaxy REST API	Galaxy CLI	Arvados GUI	Arvados REST API	Arvados CLI	CWLEXEC GUI	CWLEXEC REST API	CWLEXEC CLI
Add new workflow[2]	–	–	+	+	∅	+	∅	∅	+	∅	+	+	∅	+	+	+	+	∅	+	+	+	∅	∅	+

Feature	Airflow & CWL-Airflow			Rabix			Toil			Cromwell			REANA			Galaxy			Arvados			CWLEXEC		
Set workflow inputs[3]	–	+	+	+	∅	+	∅	∅	+	∅	+	+	∅	+	+	+	+	∅	+	+	+	∅	∅	+
Start/stop workflow execution	+	+	+	+	∅	+	∅	∅	+	∅	+	+	∅	+	+	+	+	∅	+	+	+	∅	∅	+
Manage workflow execution process[4]	+	+	+	–	∅	–	∅	∅	+	∅	–	–	∅	+	+	+	+	∅	–	+	+	∅	∅	+
Get execution results of the specific workflow[5]	+	–	–	+	∅	–	∅	∅	+	∅	+	–	∅	+	+	+	+	∅	+	+	+	∅	∅	–
View workflow execution logs	+	–	+	+	∅	+	∅	∅	+	∅	+	+	∅	+	+	+	+	∅	+	+	+	∅	∅	+
View workflow execution history and statistics	+	+	+	–	∅	–	∅	∅	+	∅	+	–	∅	+	+	+	+	∅	+	+	+	∅	∅	+

+, present; –, absent; ∅, No GUI; GUI, graphical user interface; CLI, command line interface; REST API, representational state transfer application program interface; WDL, workflow description language.

[1] assign workflow steps to the different pools and queues; use other resource utilization algorithms provided by the computing environment

[2] load the workflow from the file; create the workflow by combining the steps in GUI

[3] set the path to the job file; set input values through the GUI or CLI

[4] pause/resume workflow execution process; manually restart workflow steps

[5] get output file locations by the workflow id, step id, execution date or other identifiers

Abbreviations

CWL: Common Workflow Language

DAG: Directed Acyclic Graph

ChIP-Seq: Chromatin Immunoprecipitation Sequencing

GUI: Graphical User Interface

CLI: Command Line Interface

REST API: Representational State Transfer Application Program Interface

Declarations

Ethics approval and consent to participate: Not applicable.

Consent to publish: Not applicable.

Availability of data and materials: No new datasets or materials were generated. The source code is available under Apache license v2.0 (Apache-2.0) and can be downloaded from *https://barski-lab.github.io/cwl-airflow*, *http://doi.org/10.5281/zenodo.2852870* and RRID: SCR_017196.

Competing Interests: AVK and AB are co-founders of Datirium, LLC. Datirium, LLC provides bioinformatics software support services.

Funding: The project was supported in part by the Center for Clinical & Translational Research and Training (NIH CTSA grant UL1TR001425) and by the NIH NIGMS New Innovator Award to AB (DP2GM119134). The funders had no role in study design, data collection and analysis, decision to publish nor preparation of the manuscript.

Author contributions statement: AVK and AB conceived the project; AVK and MK wrote the software; and MK, AVK and AB wrote and reviewed the manuscript.

Acknowledgements: The authors thank all members of the CWL working group for their support and Shawna Hottinger for editorial assistance.

References

1. Leipzig J. A review of bioinformatic pipeline frameworks. Brief Bioinform. 2017;18:530–6.

2. Existing Workflow Systems [Internet]. Available from: https://s.apache.org/existing-workflow-systems

3. Common Workflow Language [Internet]. Available from: http://www.commonwl.org/

4. Why Docker? [Internet]. Available from: https://www.docker.com/why-docker

5. Kurtzer GM, Sochat V, Bauer MW. Singularity: Scientific containers for mobility of compute. PLoS One. 2017;12.

6. Amstutz P, Crusoe MR, Tijanić N, Chapman B, Chilton J, Heuer M, et al. Common Workflow Language, v1.0 [Internet]. Doi.Org. 2016. p. Available from: https://www.commonwl.org/v1.0/Workflow.html

7. Kaushik G, Ivkovic S, Simonovic J, Tijanic N, Davis-Dusenbery B, Kural D. RABIX: an open-source workflow executor supporting recomputability and interoperability of workflow descriptions. Pac Symp Biocomput. 2016;22:154–65.

8. Arvados [Internet]. Available from: https://arvados.org/

9. Giardine B, Riemer C, Hardison RC, Burhans R, Elnitski L, Shah P, et al. Galaxy: a platform for interactive large-scale genome analysis. Genome Res. 2005;15:1451–5.

10. Vivian J, Rao A, Nothaft FA, Ketchum C, Armstrong J, Novak A, et al. Rapid and efficient analysis of 20,000 RNA-seq samples with Toil. bioRxiv. 2016;2:062497.

11. Airflow [Internet]. Available from: http://airflow.incubator.apache.org/

12. Hindman B, Konwinski A, Zaharia M, Ghodsi A, Joseph AD, Katz R, et al. Mesos: A platform for fine-grained resource sharing in the data center. Proc 8th USENIX Conf Networked Syst Des Implement. 2011;295.

13. Celery Project [Internet]. Available from: http://www.celeryproject.org/

14. Barski Lab ChIP-Seq SE Workflow [Internet]. Available from: https://barski-lab.github.io/cwl-airflow/#running-sample-chip-seq-se-workflow

15. Kartashov A V, Barski A. BioWardrobe: an integrated platform for analysis of epigenomics and transcriptomics data. Genome Biol. 2015;16:158.

16. Vallabh S, Kartashov A V., Barski A. Analysis of ChIP-Seq and RNA-Seq Data with BioWardrobe. Methods Mol Biol. 2018;1783:343–60.

17. Langmead B, Trapnell C, Pop M, Salzberg SL. Ultrafast and memory-efficient alignment of short DNA sequences to the human genome. Genome Biol. 2009;10:R25.

18. Li H, Handsaker B, Wysoker A, Fennell T, Ruan J, Homer N, et al. The Sequence Alignment/Map format and SAMtools. Bioinformatics. 2009;25:2078–9.

19. Zhang Y, Liu T, Meyer CA, Eeckhoute J, Johnson DS, Bernstein BE, et al. Model-based analysis of ChIP-Seq (MACS). Genome Biol. 2008/09/19. 2008;9:R137.

20. FASTX Toolkit [Internet]. Available from: http://hannonlab.cshl.edu/fastx_toolkit/index.html

21. Barnett DW, Garrison EK, Quinlan AR, Stromberg MP, Marth GT. BamTools: a C++ API and toolkit for analyzing and managing BAM files. Bioinformatics. 2011;27:1691–2.

22. Hnisz D, Abraham BJ, Lee TI, Lau A, Saint-André V, Sigova A a, et al. Super-enhancers in the control of cell identity and disease. Cell. 2013;155:934–47.

23. Karimi K, Fortriede JD, Lotay VS, Burns KA, Wang DZ, Fisher ME, et al. Xenbase: a

genomic, epigenomic and transcriptomic model organism database. Nucleic Acids Res. 2018;46:D861–8.

24. Barski Lab CWL Workflows on GitHub [Internet]. Available from: https://github.com/Barski-lab/workflows

25. O'Connor BD, Yuen D, Chung V, Duncan AG, Liu XK, Patricia J, et al. The Dockstore: enabling modular, community-focused sharing of Docker-based genomics tools and workflows. F1000Research. 2017;6:52.

26. Landt SG, Marinov GK, Kundaje A, Kheradpour P, Pauli F, Batzoglou S, et al. ChIP-seq guidelines and practices of the ENCODE and modENCODE consortia. Genome Res. 2012;22:1813–31.

27. ENCODE ChIP-Seq pipeline [Internet]. Available from: https://github.com/ENCODE-DCC/pipeline-container

28. GA4GH-DREAM Workflow Execution Challenge [Internet]. Available from: https://www.synapse.org/#!Synapse:syn8507133/wiki/415976

Figure 1

Click here to access/download;Figure;figure_1.png

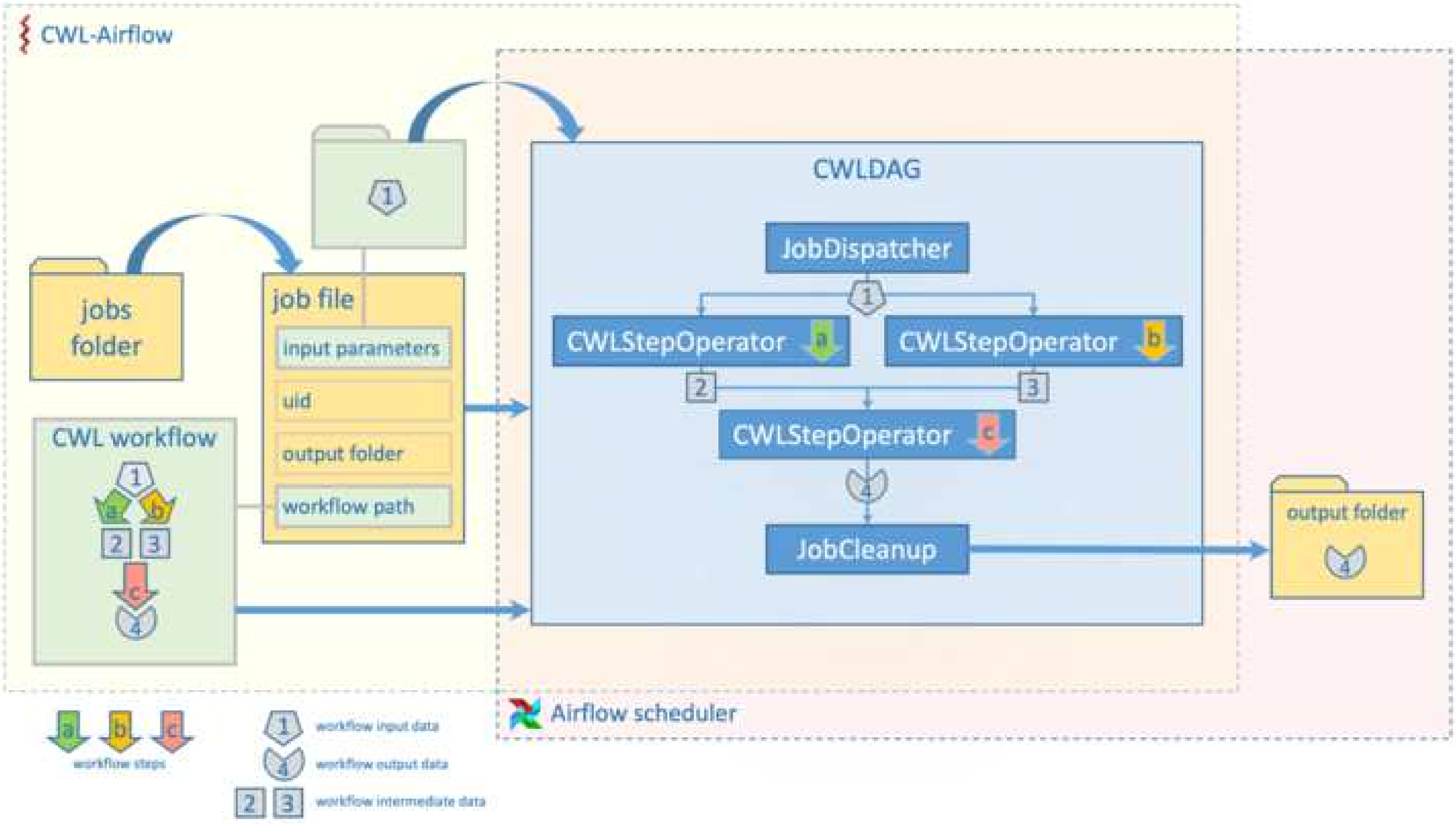

Figure 2 Click here to access/download;Figure;figure_2.png

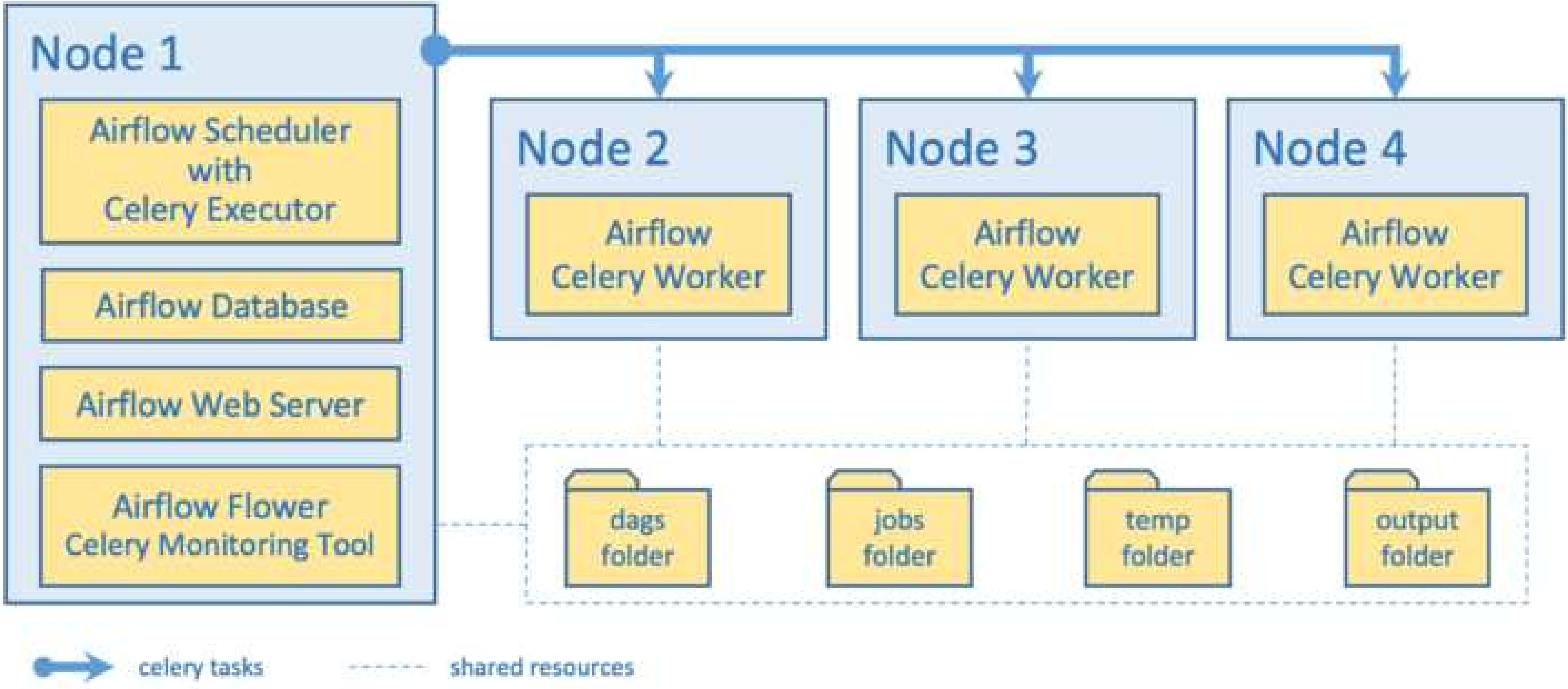

Figure 3 Click here to access/download;Figure;figure_3.png

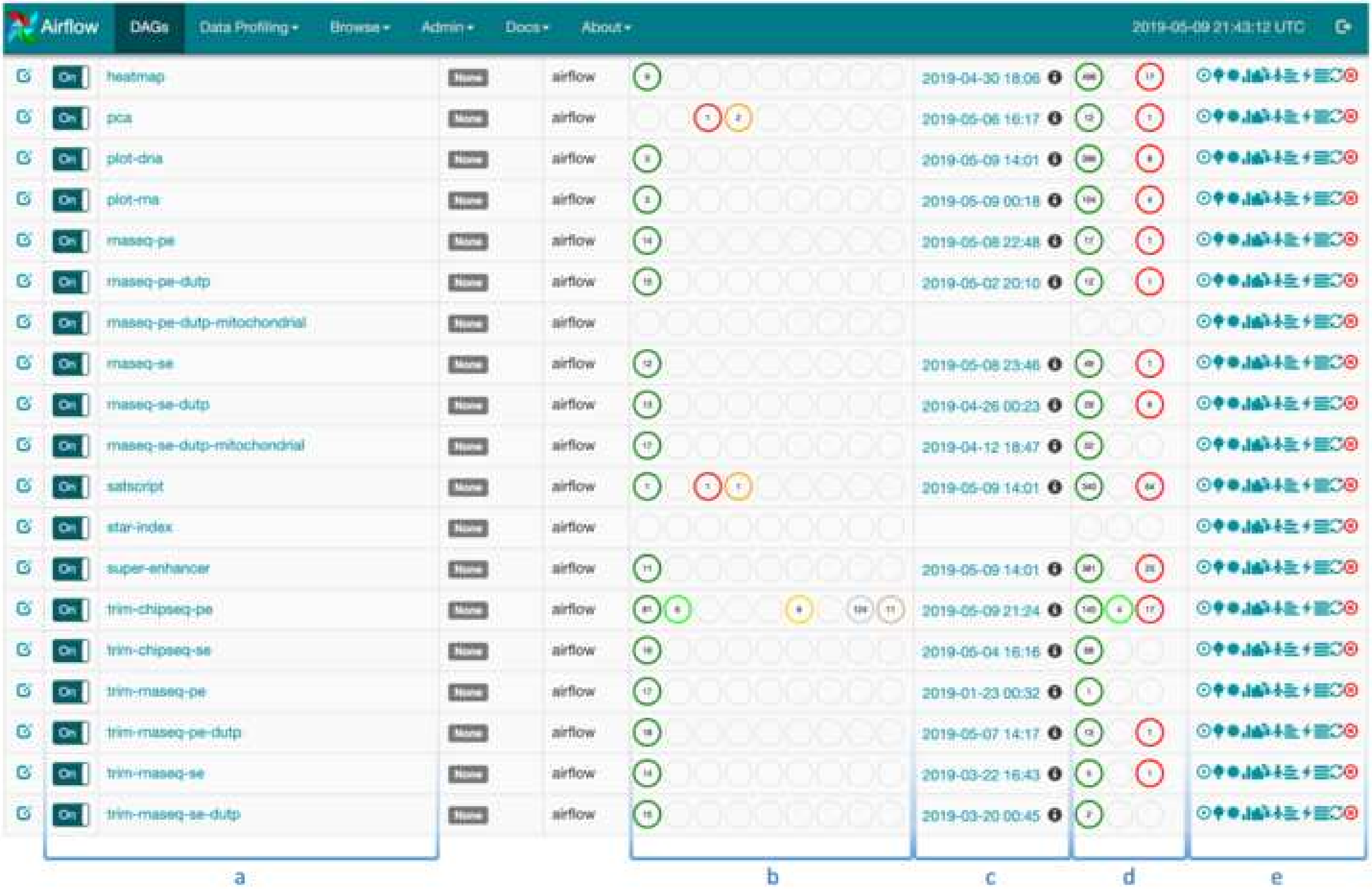

Figure 4

Click here to access/download;Figure;figure_4.png

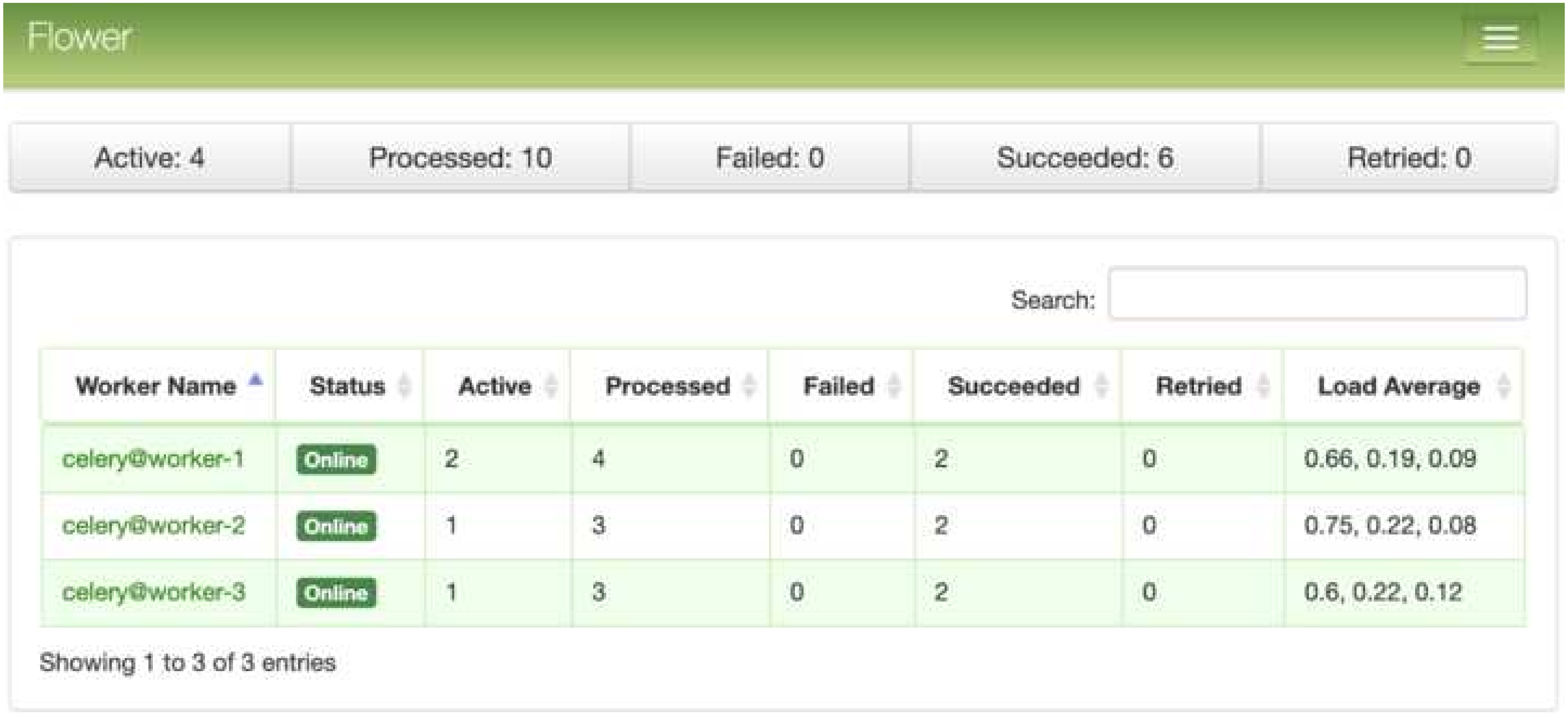

Figure 5

Click here to access/download;Figure;figure_5.png

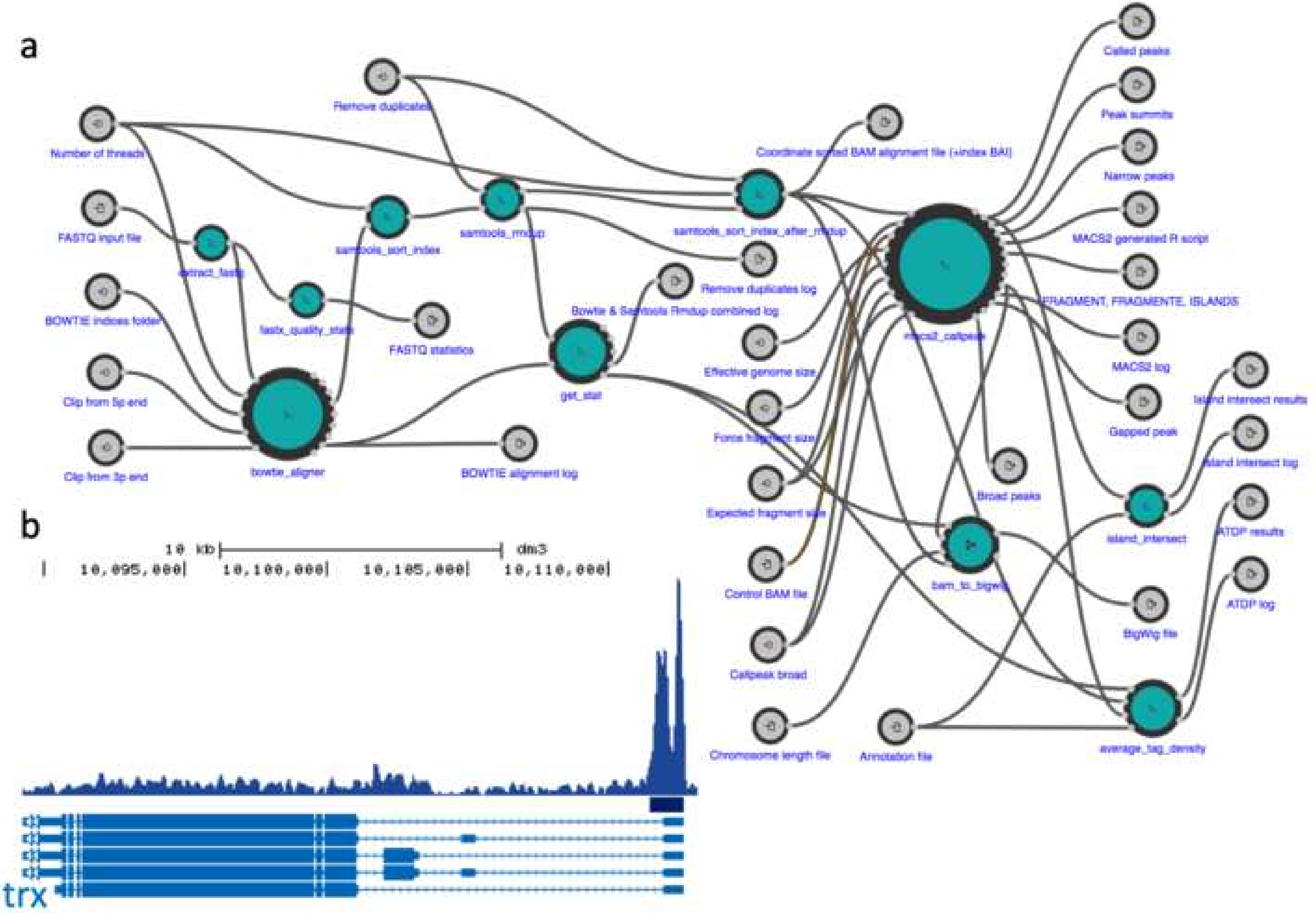

Response to reviewers

Click here to access/download
Supplementary Material
for_reviewers.docx

Personal Cover

Click here to access/download;Personal Cover;coverGigaRe.docx

Cincinnati Children's
changing the outcome together

UNIVERSITY OF Cincinnati

Allergy & Immunology

Marc E. Rothenberg, MD, PhD
Professor
Director, Division of Allergy & Immunology
Director, Cincinnati Center for Eosinophilic Disorders
Phone 513-803-0257, Fax 513-636-3310
rothenberg@cchmc.org

Amal H. Assa'ad, MD
Professor
Associate Director, Division of Allergy & Immunology
Director, Allergy & Immunology Clinical Services
Phone 513-636-7944, Fax 513-636-5835
amal.assa'ad@cchmc.org

Simon P. Hogan, PhD
Associate Professor
Director, Allergy & Immunology Research
Phone 513-636-6620, Fax 513-636-3310
simon.hogan@cchmc.org

Kimberly A. Risma, MD, PhD
Associate Professor
Director, Allergy & Immunology Fellowship Program
Phone 513-803-4230, Fax 513-636-3310
kimberly.risma@cchmc.org

J. Pablo Abonia, MD
Associate Professor

Artem Barski, PhD
Associate Professor

Sandy Durrani, MD
Assistant Professor

Thomas J. Fischer, MD
Adjunct Professor

Patricia C. Fulkerson, MD, PhD
Assistant Professor

Michelle B. Lierl, MD
Staff Physician

Andrew W. Lindsley, MD, PhD
Instructor

Stephanie L. Logsdon, MD
Assistant Professor

Ting Wen, PhD
Instructor

Nives Zimmermann, MD
Associate Professor

Kevin Titus, MBA
Business Director

Melissa Mingler, MS, MBA
Business Manager

May 21, 2019

Scott Edmunds, Ph. D.

Executive Editor,

GigaScience

Oxford University Press

Dear Dr. Edmunds,

We are re-submitting our manuscript titled "CWL-Airflow: a lightweight pipeline manager supporting Common Workflow Language" for your consideration for publication in GigaScience as a "Technology Note". We were heartened by the enthusiastic reviews of our original manuscript and hope that the updated version meets the GigaScience requirements for publication.

As suggested by reviewers, we have enhanced our manuscript in several areas: (i) We added a use case for CWL-Airflow for a multi node configuration in the Results section; (ii) We enhanced the Discussion section and improved Table 2 that compares CWL-Airflow with alternative pipeline managers. (iii) We have improved the github page for CWL-Airflow and added a CWL-Airflow virtual machine. Additionally, we have registered our software with SciCrunch and include the Research Object for our CWL workflow as a supplemental file.

Given the strong interest in CWL among computational biologists, we hope that our manuscript and CWL-Airflow will be of high interest to the readership of GigaScience.

Sincerely yours,

Artem Barski,
Associate Professor,
Divisions of Allergy & Immunology and Human Genetics
Director, Epigenomics Data Analysis Core
Cincinnati Children's Hospital Medical Center
University of Cincinnati College of Medicine

S.6.409 MLC7028,
240 Albert Sabin Way,
Cincinnati, OH 45229-3039
Phone: (513) 636-1851
Fax: (513) 636-3310
artem.barski@cchmc.org
