# Supplementary material for: CWL-Airflow: a lightweight pipeline manager supporting Common Workflow Language
Source: Gigascience. 2019 Jul 18;8(7):giz084. doi: 10.1093/gigascience/giz084 (PMC6639121; doi:10.1093/gigascience/giz084)
Supplement: giz084_Response_to_Reviewer_Comments_Original_Submission [file giz084_response_to_reviewer_comments_original_submission.pdf]

Author's Response To Reviewer Comments

Close

See the file attached as Supplementary Material.

Close
