# Supplementary material for: CWL-Airflow: a lightweight pipeline manager supporting Common Workflow Language
Source: Gigascience. 2019 Jul 18;8(7):giz084. doi: 10.1093/gigascience/giz084 (PMC6639121; doi:10.1093/gigascience/giz084)
Supplement: giz084_Response_to_Reviewer_Comments_Revision_1 [file giz084_response_to_reviewer_comments_revision_1.pdf]

Author's Response To Reviewer Comments

Close

As suggested by reviewer 2, we have corrected the typos in the text. We would also like to include a Research Object bundle for our CWL workflow as a supplemental file; however the EM system unzips the RO bundle. I'll be happy to submit it by email.

Close
