# Supplementary material for: CWL-Airflow: a lightweight pipeline manager supporting Common Workflow Language
Source: Gigascience. 2019 Jul 18;8(7):giz084. doi: 10.1093/gigascience/giz084 (PMC6639121; doi:10.1093/gigascience/giz084)
Supplement: giz084_Reviewer_1_Report_Original_Submission — Niels Drost -- 3/20/2019 Reviewed [file giz084_reviewer_1_report_original_submission.pdf]

Reviewer Report

Title: CWL-Airflow: a lightweight pipeline manager supporting Common Workflow Language

Version: Original Submission **Date:** 3/20/2019

Reviewer name: Niels Drost

Reviewer Comments to Author:

This paper describes, in a clear and concise manner, a CWL extension to the AirFlow system. It is pleasant to read, and clearly describes the advantages of CWL-Airflow over using AirFlow directly, or the use of an alternative CWL implementation.
One area where the paper can be improved is the results section. Although (as the authors write) CWL-Airflow has the advantage of running pipelines across multiple nodes, the examples shown here seem to only make use of a single node. Other advantages such as a web interface are also not shown or mentioned in the example. In this simple case (from a system point of view), the CWL reference runner would be sufficient. I advice the authors to also show a few more advanced features of CWL-Airflow in the example, e.g. by extending table 1 with a multi node run, and by adding a screenshot of the web interface.
Table 2 lists a number of advantages CWL-Airflow has over alternatives. It strikes me as odd that CWl-Airflow lists "yes" for each and every feature mentioned in the table. I encourage the authors to also find and list a few defining features of the alternative workflow management systems not (yet) supported by CWL-Airflow.

Level of Interest

Please indicate how interesting you found the manuscript: Choose an item.

Quality of Written English

Please indicate the quality of language in the manuscript: Choose an item.

Declaration of Competing Interests

Please complete a declaration of competing interests, considering the following questions:

- Have you in the past five years received reimbursements, fees, funding, or salary from an organisation that may in any way gain or lose financially from the publication of this manuscript, either now or in the future?
- Do you hold any stocks or shares in an organisation that may in any way gain or lose financially from the publication of this manuscript, either now or in the future?
- Do you hold or are you currently applying for any patents relating to the content of the manuscript?

- Have you received reimbursements, fees, funding, or salary from an organization that holds or has applied for patents relating to the content of the manuscript?
- Do you have any other financial competing interests?
- Do you have any non-financial competing interests in relation to this paper?

If you can answer no to all of the above, write 'I declare that I have no competing interests' below. If your reply is yes to any, please give details below.

I declare that I have no competing interests

I agree to the open peer review policy of the journal. I understand that my name will be included on my report to the authors and, if the manuscript is accepted for publication, my named report including any attachments I upload will be posted on the website along with the authors' responses. I agree for my report to be made available under an Open Access Creative Commons CC-BY license (http://creativecommons.org/licenses/by/4.0/). I understand that any comments which I do not wish to be included in my named report can be included as confidential comments to the editors, which will not be published.

I agree to the open peer review policy of the journal

To further support our reviewers, we have joined with Publons, where you can gain additional credit to further highlight your hard work (see: https://publons.com/journal/530/gigascience). On publication of this paper, your review will be automatically added to Publons, you can then choose whether or not to claim your Publons credit. I understand this statement.

Yes Choose an item.
