# Supplementary material for: CWL-Airflow: a lightweight pipeline manager supporting Common Workflow Language
Source: Gigascience. 2019 Jul 18;8(7):giz084. doi: 10.1093/gigascience/giz084 (PMC6639121; doi:10.1093/gigascience/giz084)
Supplement: giz084_Reviewer_2_Report_Original_Submission — Samuel Lampa, PhD -- 4/4/2019 Reviewed [file giz084_reviewer_2_report_original_submission.pdf]

Reviewer Report

Title: CWL-Airflow: a lightweight pipeline manager supporting Common Workflow Language

Version: Original Submission **Date:** 4/4/2019

Reviewer name: Samuel Lampa, PhD

Reviewer Comments to Author:

The paper on CWL-Airflow is easy to read and follow, and presents the developed functionality, a tool that enables support for Common Workflow Language (CWL) in the well established Airflow workflow manager.
Given the high recent interest in CWL, and the well established user base of the open source Airflow workflow manager, this development should be interesting and useful for many readers of GigaScience.
I have got one major issue with the manuscript, a few smaller (but important) issues, and a list of minor corrections:
Major issue: More throrough discussion
To start with, while the paper is clear and easy to follow, I find the discussion motivating this specific tool compared to other CWL managers quite thin, and leaving holes in an otherwise easy to follow thread through the paper
While motivation for the use of CWL is provided, and a table (Table 2) comparing CWL-Airflow with other CWL-based workflow managers, this information is almost completely absent in the text. Both in the main text and the table descriptions (which are absent as far as I can see?).
To be more concrete, what is available is this text (in the background)
> Unlike many of the more complicated platforms, Airflow imposes little
> overhead, is easy to install, and can be used to run task-based workflows in
> various environments ranging from standalone desktops and servers to Amazon
> or Google cloud platforms. It also scales horizontally on clusters managed
> by Apache Mesos [7] and may be configured to send tasks to Celery
> (http://www.celeryproject.org) task queue
This is OK as a start, but lacks concrete comparisons in terms of features, capabilities, compared with explicitly pointed out other tools.
Thus, the main text needs a few paragraphs leading the reader through the most important comparisons with other CWL-based tools in Table 2, giving some examples of concrete features available/not available in other tools. E.g., the text above in the background could be expanded, and then linking back and recapping on that in the discussion.
In fact, there could be one more motivating factor for introducing CWL support in Airflow that wasn't even mentioned here that the authors might find worth considering;

The fact that CWL allows defining dependencies in terms of data, while Airflow itself (at least in earlier versions, so needs double-checking) only allowed defining them in terms of tasks in the main workflow definition, which could lead to less composable workflows. CWL support should fix that, as CWL defines dependencies in terms of data. This problem in was discussed in a blog post that has circulated in the workflow community:
https://bionics.it/posts/workflows-dataflow-not-task-deps

Smaller (but important) issues

- There are multiple web links which should be linked as proper references (The first in Background, row 11)
- Figure 1 needs a few sentences describing what the figure shows.
- Table 1 and 2 need at least basic captions.
- Table 2, table-row 4 (document rows 14-18), table-column 6: It says CWL support in Cromwell is "planned". That doesn't seem like it should qualify to make the cell "green", if it is not yet implemented?
- Table 2, document rows 58-60, 16-19, 25-26, 45-47: The following features are unclear and need clarifications (e.g. in the missing table caption): "Workflow execution", "Cross DAG task dependencies", "Backfilling DAGs", "Implements workflow queue".

Minor corrections

- In the Abstract, under "Findings"
"on variety" should be "on a variety of"
- Background, row 22:
"CWL specification" should be "The CWL specification"
- Methods, row 31:
"(v1.0 [3])" should probably be "(v1.0) [3]"
- Methods, row 39:
"Airflow scheduler" should be "The Airflow scheduler"
- Figure 2: Text should be added as text, not as part of the bitmap image.
- Results, line 57:
"CWL-Airflow package" should be "The CWL-Airflow package"
- Section "Portability of CWL analysis"
In the section title itself, and the first sentence, I think "analysis" should be "analyses" (plural).
- Section "Portability of CWL analysis", row 17 and 24:
Add spaces before citations.

Installation issues:

For information, I got the following errors when trying to install on Python 3.7.0 on Conda, on Xubuntu 16.04:

```bash
$ pip install cwl-airflow --find-links https://michael-kotliar.github.io/cwl-airflow-wheels/
Looking in links: https://michael-kotliar.github.io/cwl-airflow-wheels/
Collecting cwl-airflow
```

Downloading
https://files.pythonhosted.org/packages/51/91/3ca7f352ed4f8ea6984481f100d3a8d9a3c7d3cdc2e443d6946c96c10fe2/cwl-airflow-1.0.14.tar.gz (6.2MB)
100% |################################| 6.3MB 4.0MB/s
Collecting cwltool==1.0.20180622214234 (from cwl-airflow)
Downloading
https://files.pythonhosted.org/packages/92/a5/d9739eb51b3e2d55438a194ef7bd7f55ae8785a0219563006fdbab37b80a/cwltool-1.0.20180622214234-py2.py3-none-any.whl (642kB)
100% |################################| 645kB 4.8MB/s
Collecting jsonmerge (from cwl-airflow)
Downloading
https://files.pythonhosted.org/packages/e7/62/fd61413785762ba311da5636a9e56ef26ecafaafdd0e7614c570857882b0/jsonmerge-1.6.0.tar.gz
Collecting apache-airflow==1.9.0 (from cwl-airflow)
Downloading
https://files.pythonhosted.org/packages/9e/12/6c70f9ef852b3061a3a6c9af03bd9dcdcaecb7d75c8898f82e3a54ad5f87/apache-airflow-1.9.0.tar.gz (2.4MB)
100% |################################| 2.4MB 2.6MB/s
Complete output from command python setup.py egg_info:
Traceback (most recent call last):
File "<string>", line 1, in <module>
File "/tmp/pip-install-pbyn1rcm/apache-airflow/setup.py", line 102
async = [
^
SyntaxError: invalid syntax
```
I had better luck using Python 2.7.14 via PyEnv, on the same Operating System.
By running the following commands:
```bash
pip install cwl-airflow --find-links https://michael-kotliar.github.io/cwl-airflow-wheels
cwl-airflow init
cwl-airflow demo --auto
```
... I managed to get the Airflow web interface running, showing up three DAGs.
Three empty folders showed up in the current directory:
01a7dde7-acf0-478a-b2d5-6522538a26db
559a2084-dc91-4524-bf1a-745f7dd801e6
baeaee20-9872-4aeb-bca0-6dfeaba9737d
I did not manage to get any workflows to run in the time I was able to spend trying.
In other words, it looked like this screenshot, except no green circles in "Recent Tasks" or "DAG runs":
https://raw.githubusercontent.com/Barski-lab/cwl-airflow/master/docs/screen.png
Thus, I was not able to verify that the tool works.
```

One way the authors could help us reviewers verify this, would be to provide a virtual machine (exported as an .ova file) with everything pre-installed, and a shell script to execute, with information about what information to exect.
I would actually strongly suggest doing that, as that, and uploading the image to Zenodo or Figshare to get a persistent DOI, as this would provide a relatively fool-proof way to make sure that the tool can be tried out, tested, and learned, without extensive troubleshooting.

Level of Interest

Please indicate how interesting you found the manuscript: Choose an item.

Quality of Written English

Please indicate the quality of language in the manuscript: Choose an item.

Declaration of Competing Interests

Please complete a declaration of competing interests, considering the following questions:

- Have you in the past five years received reimbursements, fees, funding, or salary from an organisation that may in any way gain or lose financially from the publication of this manuscript, either now or in the future?
- Do you hold any stocks or shares in an organisation that may in any way gain or lose financially from the publication of this manuscript, either now or in the future?
- Do you hold or are you currently applying for any patents relating to the content of the manuscript?
- Have you received reimbursements, fees, funding, or salary from an organization that holds or has applied for patents relating to the content of the manuscript?
- Do you have any other financial competing interests?
- Do you have any non-financial competing interests in relation to this paper?

If you can answer no to all of the above, write 'I declare that I have no competing interests' below. If your reply is yes to any, please give details below.

I declare that I have no competing interests

I agree to the open peer review policy of the journal. I understand that my name will be included on my report to the authors and, if the manuscript is accepted for publication, my named report including any attachments I upload will be posted on the website along with the authors' responses. I agree for my report to be made available under an Open Access Creative Commons CC-BY license (http://creativecommons.org/licenses/by/4.0/). I understand that any comments which I do not wish to

be included in my named report can be included as confidential comments to the editors, which will not be published.

I agree to the open peer review policy of the journal

To further support our reviewers, we have joined with Publons, where you can gain additional credit to further highlight your hard work (see: https://publons.com/journal/530/gigascience). On publication of this paper, your review will be automatically added to Publons, you can then choose whether or not to claim your Publons credit. I understand this statement.

Yes Choose an item.
